# Comparative Efficacy and Safety of 0.05% Cyclosporine A and 3% Diquafosol Sodium in Dry Eye Disease: A Systematic Review and Meta-Analysis with Trial Sequential Analysis

**DOI:** 10.3390/jcm15124823

**Published:** 2026-06-21

**Authors:** Abdullah Y. Alsuhail, Abdullah M Alkandari, Ahmed Mohammad, Sara Almutawtah, Yaqoub AlFoudari, Fatmah S. Semairan, Fahad Mohammad, Abdullah AlOtaibi, Omar Almutairi, Rashed A. Alasoosi, Shahad T. Ahmad, Abdullah M. Alharran

**Affiliations:** 1 College of Medicine and Medical Sciences, Arabian Gulf University, Manama P.O. Box 26671, Bahrain; alahytm@agu.edu.bh; 2 Faculty of Medicine, The University of Jordan, Amman 11942, Jordan; abd0208469@ju.edu.jo; 3 Kuwait Institute for Medical Specializations, Kuwait City P.O. Box 13115, Kuwait; abdalhussain9115@moh.gov.kw (A.M.); sarah.almutoutah@hscm.ku.edu.kw (S.A.); yalfoudari913@moh.gov.kw (Y.A.); semairan9129@moh.gov.kw (F.S.S.); alasoosi6824@moh.gov.kw (R.A.A.); ahmad8952@moh.gov.kw (S.T.A.); 4 Royal College of Surgeons in Ireland, D02 YN77 Dublin, Ireland; fahadmohammad22@rcsi.com (F.M.); abdullahalotaibi@rcsi.com (A.A.); omaralmutairi19@rcsi.com (O.A.)

**Keywords:** Dry Eye Disease, Cyclosporine, Diquafosol, meta-analysis, tear film, ocular surface

## Abstract

**Background:** Dry Eye Disease (DED) is a multifactorial ocular surface disorder characterized by tear film instability and inflammation. Cyclosporine A, an immunomodulator, and Diquafosol sodium, a mucin secretagogue, represent two distinct therapeutic pathways. However, current evidence directly comparing their clinical efficacy is inconsistent. This meta-analysis aimed to compare treatment outcomes and efficacy between 0.05% Cyclosporine A and 3% Diquafosol sodium in patients with moderate-to-severe DED. **Methods:** In January 2026, we conducted a systematic search of PubMed, Scopus, Web of Science, and the Cochrane Library for randomized controlled trials directly comparing 0.05% Cyclosporine A to 3% Diquafosol sodium in adult patients with moderate-to-severe DED. For the meta-analysis, we used R 4.5.0 with R Studio 2024.12.1+563. **Results:** We included six RCTs with a total of 859 patients. No significant differences were found between Cyclosporine A and Diquafosol sodium in Tear Break-Up Time (TBUT) at 4, 8, or 12 weeks. Cyclosporine A showed a suggestive greater improvement in Schirmer test scores at 4 weeks (SMD = 0.35, 95% CI 0.07 to 0.63). A modest benefit in symptom scores favoring Diquafosol sodium was observed at 12 weeks (SMD = 0.23, 95% CI 0.06 to 0.41). Subgroup analysis suggested this symptomatic benefit may be more pronounced in patients with severe disease, although subgroup interaction tests were not statistically significant. There were no significant differences in corneal or conjunctival staining at any time point. The risk of adverse events did not differ significantly between treatments. **Conclusions:** Early improvement in tear production showed a potential benefit for Cyclosporine A, while longer-term symptomatic relief showed a potential benefit for Diquafosol sodium, with suggestive evidence in severe disease. However, these findings should be interpreted cautiously, given the methodological limitations and inconclusive TSA evidence for several outcomes. Future large-scale, standardized trials with extended follow-up are warranted to confirm these findings.

## 1. Introduction

Dry Eye Disease (DED) is a multifactorial disease that evolved from being considered as a simple eye irritant to an increasingly growing inflammatory condition that affects millions of people around the world [1]. According to the TFOS DEWS II definition, DED is a disease of the ocular surface characterized by a loss of homeostasis of the tear film, where tear film instability, hyperosmolarity, and ocular surface inflammation play critical etiological roles [1,2]. This inflammation initiates a vicious cycle that persists, where dry surfaces cause the release of inflammatory cytokines, which further damage the lacrimal functional unit and worsens tear deficiency [3]. If left untreated, severe and chronic dryness can cause corneal damage, ulceration, and vision loss, resulting in a negative economic impact by reducing individual productivity and overall quality of life [4]. While traditional lubricants remain the traditionally used line of treatment for DED patients, the chronic inflammatory characteristics of the disease, which can cause vision impairment, have prompted clinicians to move beyond palliative lubrication toward more targeted pharmaceutical interventions [5].

Cyclosporine A is a calcineurin inhibitor that suppresses T-cell-mediated inflammation. By reducing the expression of inflammatory markers such as HLA-DR, it allows the lacrimal gland and conjunctival goblet cells to gradually recover their natural function over time [6,7]. Diquafosol sodium is a mucin secretagogue that agonistically interacts with the ocular surface’s P2Y2 receptor. It increases the production of tear fluid from goblet cells and lacrimal glands and promotes the secretion of mucin. In DED, this mechanism is thought to increase tear film stability [8,9].

Currently, there is no clear gold standard for how to treat individuals with DED [10]. The current clinical evidence remains insufficient for the two most important objective markers for DED: Tear Film Break-Up Time (TBUT) and Schirmer test scores [11]. Previous randomized controlled trials suggest that Diquafosol’s mucin-boosting properties lead to better short-term TBUT results [12,13], while other studies indicate that the anti-inflammatory power of Cyclosporine provides more robust, long-term improvement in tear volume as measured by the Schirmer test [14]. Consequently, this inconsistency in the findings of available trials with different disease durations and baseline disease severity across studies undermines the ability to draw definitive, evidence-based recommendations favoring one agent over the other.

Because the current body of literature provides multiple trials comparing Cyclosporine A to Diquafosol with variable findings, a rigorous analysis is warranted to address the clinically relevant gap in the evidence base surrounding the optimal pharmacologic management of DED. It is hypothesized that the difference in their mechanisms of action might result in different outcomes in this group of patients. Therefore, our systematic review and meta-analysis aims to provide recent comprehensive evidence regarding the efficacy of 0.05% Cyclosporine A compared to 3% Diquafosol in patients with DED. We specifically focused on 0.05% Cyclosporine A and 3% Diquafosol sodium as they are the most widely studied concentrations in the literature, both are the standard clinically available doses approved for DED treatment in multiple countries.

## 2. Methods

### 2.1. Study Registration

The study protocol was registered on PROSPERO (CRD420261306828). This systematic review adhered to the Preferred Reporting Items for Systematic Reviews and Meta-Analyses (PRISMA) statement standards [15] and was conducted in accordance with the Cochrane Handbook for Systematic Reviews [16]. The PRISMA checklist is provided in the supplementary appendix (Table S2).

### 2.2. Literature Search and Study Selection

We searched PubMed, Scopus, Web of Science, and the Cochrane Library from inception up to January 2026 using search terms (“dry eye*” OR “keratoconjunctivitis sicca” OR “ocular surface disease”) AND (cyclosporin* OR CsA OR restasis OR “Immunosuppressive eye drops” OR Ikervis) AND (Diquafosol OR “P2Y2 receptor agonist” OR diquas OR INS365 OR “INS 365”). The literature search was conducted without language restrictions at the database level. However, non-English studies were excluded during the screening phase due to the absence of translation resources. Detailed search strategies are displayed in Table S1. References were uploaded to Rayyan (Qatar Computing Research Institute, Doha, Qatar) for screening [17]. Two independent reviewers conducted a two-step screening including Title/abstract screening and full-text screening for final eligibility. Disagreements were resolved by a third reviewer.

### 2.3. Eligibility Criteria

We included studies meeting the following PICO criteria:

**Population:** Adult patients (≥18 years) with moderate or severe Dry Eye Disease (DED).

**Intervention:** 3% Diquafosol sodium eye drops.

**Comparator:** 0.05% Cyclosporine A eye drops.

**Outcomes:** At least one of the following: Tear Break-Up Time (TBUT), Corneal staining scores, and Schirmer test results.

**Study Design:** Randomized controlled trials (RCTs).

We excluded reviews, observational studies, non-English studies, in vitro/animal studies, case reports, and non-peer-reviewed publications.

### 2.4. Data Extraction

Two independent reviewers extracted data using a standardized Excel sheet, including:

**Study characteristics:** Study design, population, sample size, intervention regimen, follow-up period, and key findings.

**Baseline characteristics:** Age, sex, TBUT, Schirmer test, corneal staining scores, and OSDI.

**Outcome measurements:** TBUT, corneal/conjunctival staining, Schirmer test, and adverse events.

### 2.5. Quality Assessment

Two independent reviewers assessed the risk of bias in included RCTs using the Cochrane Risk of Bias (ROB2) tool [18]. This evaluation encompassed an assessment of the randomization process, concealment of the allocation sequence, deviations from the intended interventions, utilization of appropriate analysis to estimate the effect of assignment to intervention, measurement of the outcome, selection of the reported results, and overall risk of bias. Any conflict was resolved by a third author. The Robvis web tool (University of Bristol, Bristol, UK) was used to create quality assessment figures [19].

### 2.6. Outcome Definition

Tear Break-Up Time (TBUT) was defined as the time in seconds between a complete blink and the appearance of the first corneal dry spot, measuring tear film stability. The Schirmer test was defined as millimeters of wetting per 5 min, providing an objective assessment of overall tear volume and aqueous production. Corneal and conjunctival staining assess the extent of ocular surface damage, with studies using different grading systems: the Oxford Grading System (0–5 scale) and the National Eye Institute (NEI) scale (0–15 scale). Symptom scores were derived from validated instruments, including the Ocular Surface Disease Index (OSDI) and the Symptom Assessment in Dry Eye (SANDE) questionnaire.

### 2.7. Statistical Analysis

We used R version 4.5.0 (R Foundation for Statistical Computing, Vienna, Austria) with R Studio version 2024.12.1+563 (Posit Software, PBC, Boston, MA, USA) [20,21]. Continuous outcomes were analyzed as standardized mean differences (SMD) with 95% confidence intervals (CI). SMD expresses the treatment effect in units of standard deviation, allowing meaningful pooling of outcomes measured on different scales. This approach preserves the direction and relative magnitude of treatment effects without requiring arbitrary scale conversions. For clinical interpretation, conventional benchmarks suggest that SMD values of 0.2, 0.5, and 0.8 represent small, moderate, and large effect sizes, respectively (Cohen’s d).

Dichotomous data were analyzed as risk ratios (RR) with 95% CI. A random-effects model was applied to all analyses. Heterogeneity was assessed using I^2^ and Chi^2^ statistics, with I^2^ of 50% or more indicating significant heterogeneity. For outcomes with substantial heterogeneity, we employed a multifaceted approach to explore and address heterogeneity: (1) leave-one-out sensitivity analyses to identify influential studies; (2) Baujat plots to visualize each study’s contribution to overall heterogeneity and pooled effect; (3) pre-specified subgroup analyses by follow-up duration and disease severity. Publication bias was assessed using Doi plots and the LFK index [22]. If asymmetry was detected, the trim-and-fill method was applied to adjust for potential missing studies [23].

Trial Sequential Analysis (TSA) was performed using TSA Viewer (Version 0.9.5.10 Beta) (Copenhagen Trial Unit, Centre for Clinical Intervention Research, Copenhagen University Hospital, Copenhagen, Denmark) [24] to control for random errors due to repeated testing, with α = 0.05, β = 0.10 (90% power), and O’Brien–Fleming spending boundaries. The required information size (RIS) was estimated without heterogeneity adjustment and increased by 3% to account for the sequential design.

## 3. Results

### 3.1. Literature Search

**Figure 1:**
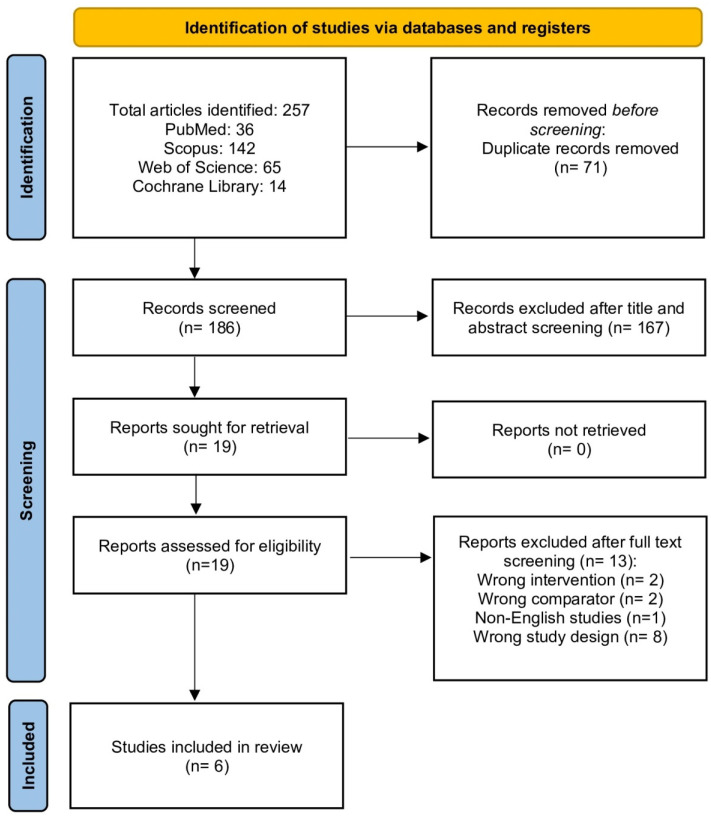
PRISMA flow chart for the systematic search and selection process.

Our search identified 257 records. After removing 71 duplicates, 186 records were screened by title/abstract. Of these, 19 full-text articles were assessed, and 13 were excluded due to wrong intervention, comparator, study design, or language. Six RCTs were included in the final analysis [25,26,27,28,29,30] (Figure 1).

### 3.2. Study Characteristics

A total of six RCTs involving 859 patients were included [25,26,27,28,29,30]. The sample sizes ranged from 40 to 282 participants. All studies compared 0.05% Cyclosporine A (twice daily) with 3% Diquafosol sodium (six times daily, except one study with twice daily [30]. Follow-up duration was 12 weeks across all trials. Baseline characteristics varied across the included studies. The mean age of participants ranged from 20.0 years (Xu et al. 2024 [25], college students) to 64.3 years (Lee et al. 2016 [27], post-cataract surgery patients). The proportion of female participants ranged from 48.3% to 100%. Baseline TBUT values were consistently low across all studies (range: 2.23–4.57 s), confirming the presence of tear film instability. Baseline Schirmer test values showed wider variation (range: 3.97–8.4 mm), with lower values indicating more severe aqueous deficiency. Corneal staining scores ranged from 1.41 to 5.68. Notably, one study (Lee et al. 2016 [27]) included patients with mild-to-moderate disease following cataract surgery, while the remaining studies enrolled patients with moderate-to-severe DED.

Detailed study and baseline characteristics are summarized in Table 1 and Table 2.

### 3.3. Quality Assessment

**Figure 2:**
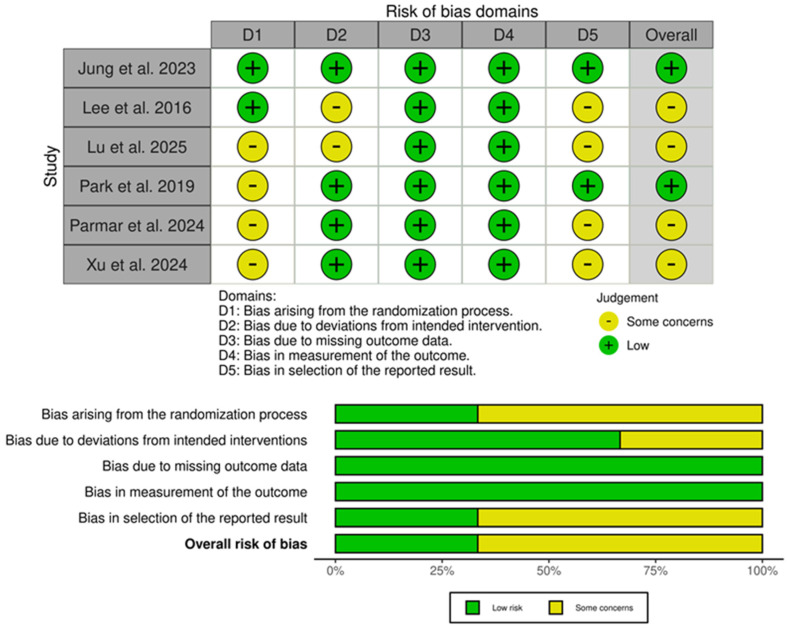
Summary and traffic light plots of the risk of bias of the included randomized controlled trials [25,26,27,28,29,30].

Using the ROB2 tool, two studies [26,29] were rated as low risk of bias, while the remaining four had some concerns or high risk [25,27,28,30], primarily due to randomization and deviation from intended interventions (Figure 2).

### 3.4. Efficacy and Safety Outcomes

#### 3.4.1. Tear Break-Up Time (TBUT)

**Figure 3:**
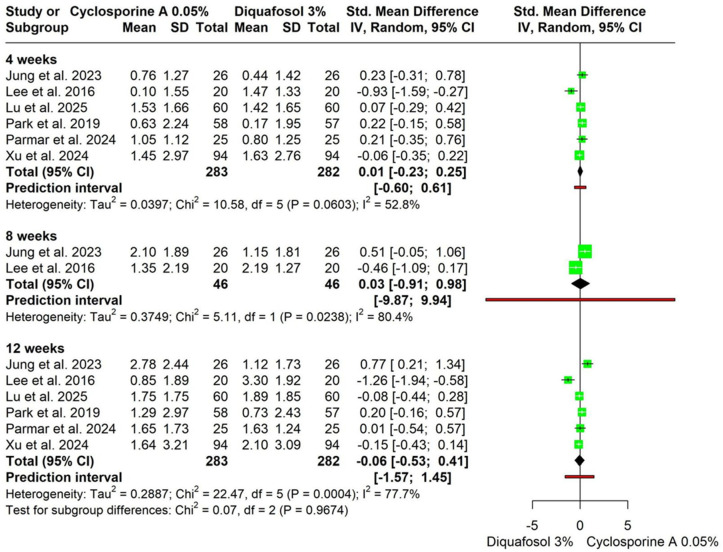
Forest plot for subgroup analysis by follow-up time for change in Tear Break-Up Time (TBUT), (sec) [25,26,27,28,29,30].

At 4 weeks, no significant difference was observed between Cyclosporine A and Diquafosol sodium (SMD = 0.01, 95% CI −0.23 to 0.25; *p* = 0.92; I^2^ = 52.8%). At 8 weeks, no significant difference was observed (SMD = 0.03, 95% CI −0.91 to 0.98; *p* = 0.95; I^2^ = 80.4%). At 12 weeks, no significant difference was observed (SMD = −0.06, 95% CI −0.53 to 0.41; *p* = 0.79; I^2^ = 77.7%). Subgroup analysis demonstrated no significant differences in treatment effects across follow-up durations (χ^2^ = 0.07, df = 2, *p* = 0.9674) (Figure 3). Heterogeneity at 4 weeks resolved after excluding Lee et al. (2016) [27], with no change in the overall effect estimate. Baujat plot identified Lee et al. as the main contributor to heterogeneity at 12 weeks, while Doi plot showed no evidence of publication bias (Figures S1–S3).

#### 3.4.2. Corneoconjunctival Fluorescein Staining

**Figure 4:**
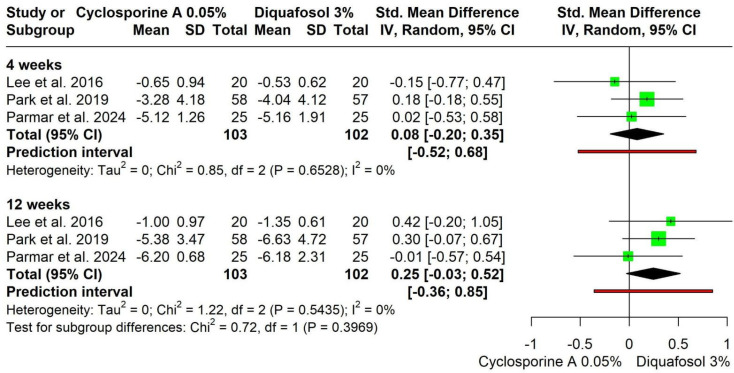
Forest plot for subgroup analysis by follow-up time for change in Corneoconjunctival Fluorescein Staining [27,29,30].

At 4 weeks, no significant difference was observed (SMD = 0.08, 95% CI −0.20 to 0.35; *p* = 0.57; I^2^ = 0%). At 12 weeks, no significant difference was observed (SMD = 0.25, 95% CI −0.03 to 0.52; *p* = 0.08; I^2^ = 0%). Subgroup analysis showed no significant difference across follow-up durations (χ^2^ = 0.72, df = 1, *p* = 0.3969) (Figure 4). Doi plot showed no asymmetry, indicating the absence of publication bias. However, Trial Sequential Analysis indicated that the current evidence is underpowered and inconclusive for this outcome (Figures S4 and S5).

#### 3.4.3. Corneal Fluorescein Staining

**Figure 5:**
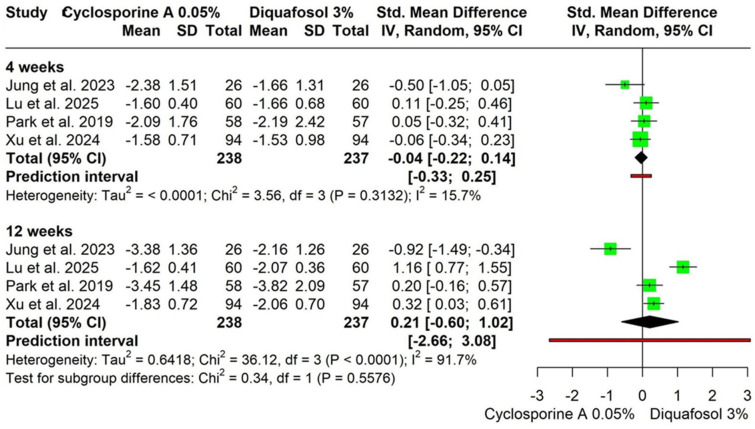
Forest plot for subgroup analysis by follow-up time for change in Corneal Fluorescein Staining [25,26,28,29].

At 4 weeks, no significant difference was observed (SMD = −0.04, 95% CI −0.22 to 0.14; *p* = 0.65; I^2^ = 15.7%). At 12 weeks, no significant difference was observed (SMD = 0.21, 95% CI −0.60 to 1.02; *p* = 0.61; I^2^ = 91.7%). Subgroup analysis showed no significant effect by follow-up duration (*p* = 0.5576) (Figure 5). High heterogeneity at 12 weeks (I^2^ = 91.7%) was primarily driven by Lu et al. (2025) [28] and Jung et al. (2023) [26], as identified by Baujat plot analysis. Doi plot showed only minor asymmetry, suggesting low risk of publication bias (Figures S6 and S7).

#### 3.4.4. Conjunctival Fluorescein Staining

**Figure 6:**
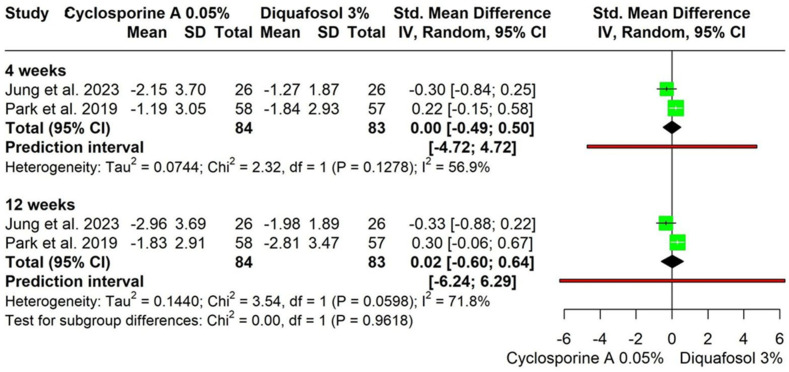
Forest plot for subgroup analysis by follow-up time for change in Conjunctival Fluorescein Staining [26,29].

At 4 weeks, no significant difference was observed (SMD = 0.00, 95% CI −0.49 to 0.50; *p* = 0.99; I^2^ = 56.9%). At 12 weeks, no significant difference was observed (SMD = 0.02, 95% CI −0.60 to 0.64; *p* = 0.95; I^2^ = 71.8%). Subgroup analysis showed no significant effect by follow-up duration (*p* = 0.9618) (Figure 6). Doi plot demonstrated major asymmetry (Figure S8).

#### 3.4.5. Schirmer Test

**Figure 7:**
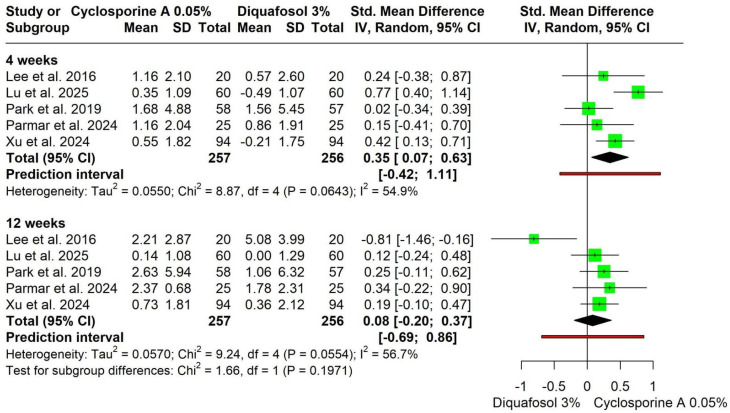
Forest plot for subgroup analysis by follow-up time for change in Schirmer Test [25,27,28,29,30].

Cyclosporine A 0.05% showed a significantly greater improvement in Schirmer test values at 4 weeks compared with Diquafosol 3% (SMD = 0.35, 95% CI 0.07 to 0.63; I^2^ = 54.9%), while no significant difference was observed at 12 weeks (SMD = 0.08, 95% CI −0.20 to 0.37; I^2^ = 56.7%), with no significant subgroup effect by follow-up duration (*p* = 0.1971) (Figure 7).

At 12 weeks, heterogeneity resolved after removing Lee et al. [27] and showed a significant effect favoring Cyclosporine A (SMD = 0.20, 95% CI 0.02 to 0.38, I^2^ = 0%) (Figure S9). Doi plot analysis showed minor asymmetry (Figure S10).

#### 3.4.6. Symptom Scores

**Figure 8:**
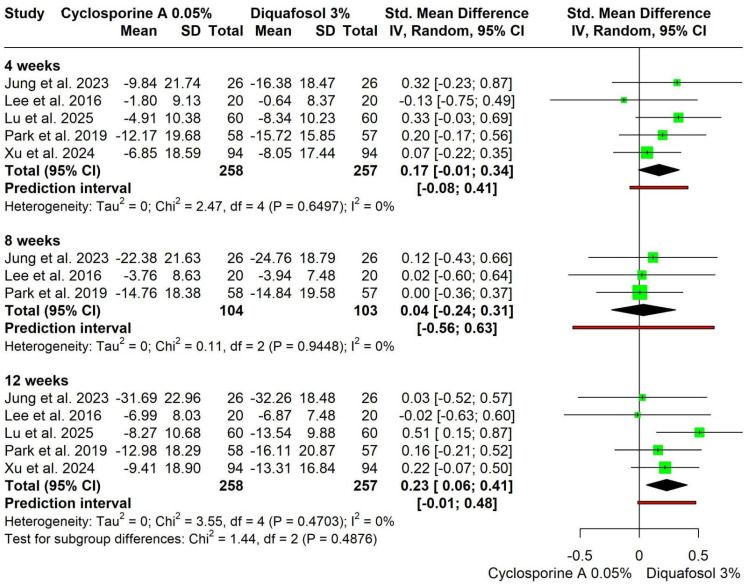
Forest plot for subgroup analysis by follow-up time for change in symptom scores [25,26,27,28,29].

No significant difference in symptom score improvement was observed between Cyclosporine A 0.05% and Diquafosol 3% at 4 weeks (SMD = 0.17, 95% CI −0.01 to 0.34; I^2^ = 0%) or 8 weeks (SMD = 0.04, 95% CI −0.24 to 0.31; I^2^ = 0%), while a small but statistically significant benefit favoring Diquafosol 3% emerged at 12 weeks (SMD = 0.23, 95% CI 0.06 to 0.41; I^2^ = 0%), with no significant subgroup differences across time points (*p* = 0.4876) (Figure 8). At 12 weeks, Doi plot analysis demonstrated minor asymmetry (Figure S11).

#### 3.4.7. Adverse Events

**Figure 9:**
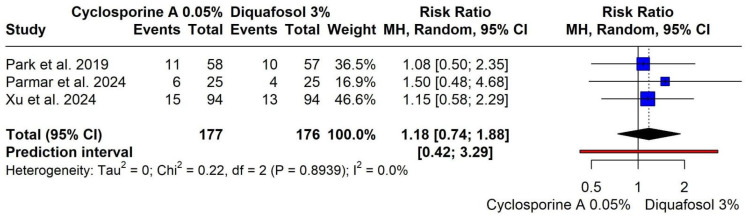
Forest plot for adverse events [25,29,30].

There was no significant difference in the risk of adverse events between Cyclosporine A 0.05% and Diquafosol 3% (RR = 1.18, 95% CI 0.74 to 1.88; I^2^ = 0.0%), with a prediction interval of 0.42 to 3.29, indicating comparable safety profiles (Figure 9). Doi plot analysis showed minor asymmetry (Figure S12).

#### 3.4.8. Subgroup Analysis by Disease Severity

##### Change in TBUT, (Sec)

**Figure 10:**
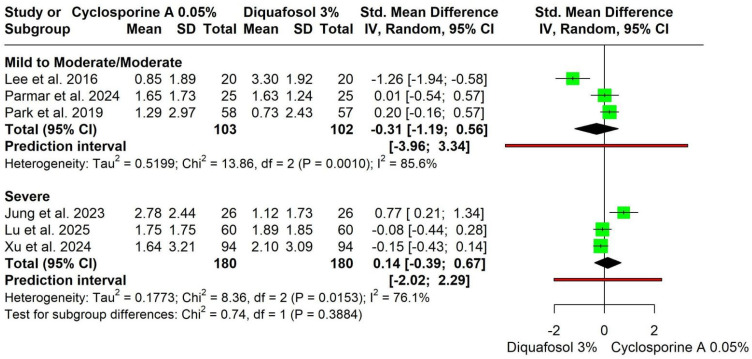
Forest plot for subgroup analysis by disease severity for change in Tear Break-Up Time (TBUT), (sec) [25,26,27,28,29,30].

Subgroup analysis by disease severity showed no significant difference between Cyclosporine A 0.05% and Diquafosol 3% in TBUT improvement among patients with mild-to-moderate/moderate disease (SMD = −0.31, 95% CI −1.19 to 0.56; I^2^ = 85.6%) or severe disease (SMD = 0.14, 95% CI −0.39 to 0.67; I^2^ = 76.1%), with no significant subgroup interaction (*p* = 0.3884) (Figure 10). Heterogeneity was not resolved with leave-one-out meta-analysis (Figure S13). Baujat plot identified Jung et al. (2023) [26] and Xu et al. (2024) [25] as principal contributors to heterogeneity. The Doi plot showed major asymmetry; however, trim-and-fill analysis imputed no missing studies, confirming robustness (Figures S13–S16).

##### Change in Schirmer Test

**Figure 11:**
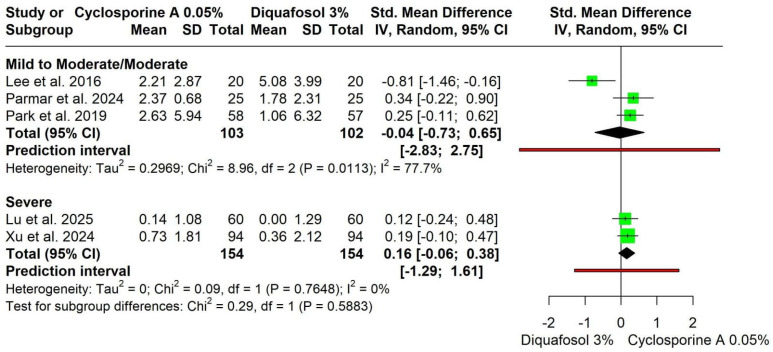
Forest plot for subgroup analysis by disease severity for change in Schirmer Test [25,27,28,29,30].

No significant differences in patients with mild-to-moderate disease (SMD = −0.04, 95% CI −0.73 to 0.65; I^2^ = 77.7%) or severe disease (SMD = 0.16, 95% CI −0.06 to 0.38; I^2^ = 0%), with no significant subgroup interaction (*p* = 0.5883) (Figure 11). Heterogeneity in the mild-to-moderate subgroup resolved after excluding Lee et al. (2016) [27]. The Doi plot showed minor asymmetry (Figures S17 and S18).

##### Change in Symptom Scores

**Figure 12:**
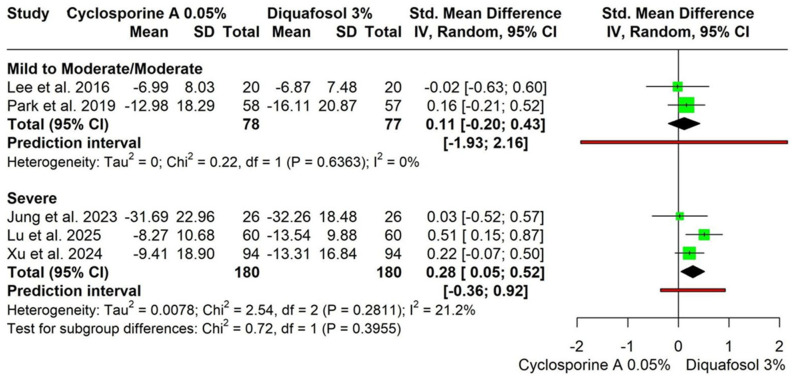
Forest plot for subgroup analysis by disease severity for change in symptom scores [25,26,27,28,29].

No significant difference in patients with mild-to-moderate disease (SMD = 0.11, 95% CI −0.20 to 0.43; I^2^ = 0%). A small benefit favoring Diquafosol sodium was observed in severe disease (SMD = 0.28, 95% CI 0.05 to 0.52; I^2^ = 21.2%); however, the subgroup interaction was not statistically significant (*p* = 0.3955) (Figure 12). The Doi plot showed no asymmetry (Figure S19).

## 4. Discussion 

### 4.1. Summary of Findings 

This comprehensive systematic review and meta-analysis compared Cyclosporine A with Diquafosol in patients with DED across multiple clinical outcomes. According to our findings, there were no significant differences between the two therapeutic medications during different follow-up durations in TBUT, corneal and Conjunctival Fluorescein Staining, Schirmer test, symptom score, and adverse events. However, we found minor yet significant differences favoring Cyclosporine in the Schirmer test on the 4-week follow-up while the Diquafosol group had significantly improved symptom score at the 12- week follow-up. Regarding disease severity outcomes, results demonstrated a significant change in symptom scores favoring the Diquafosol group with a mean difference of 0.28 in patients with severe disease. Other findings did not demonstrate any significant superiority between the two groups.

### 4.2. Interpretation of Findings 

Schirmer test and TBUT were proven to provide the objective, measurable data needed to distinguish between different subtypes of DED. While the Schirmer test is implemented by measuring tear volume and production, TBUT evaluates tear stability by measuring the interval between a complete blink and the appearance of the first dry spot on the corneal surface [31,32]. Our results showed that Cyclosporine A facilitates a substantial early enhancement in Schirmer test scores, which aligns with its recognized function in reestablishing the lacrimal functional unit [33]. Wan et al. [14] indicated that lowering T-cell mediated inflammation in the lacrimal gland allows neuronal sensitivity and reflex tearing to function normally, which results in an early increase in aqueous production in some groups. Diquafosol works differently by increasing the movement of fluids over the conjunctival surface instead of directly increasing the lacrimal gland’s ability to make tears [34].

The modest symptomatic advantage observed with Diquafosol at 12 weeks reached statistical significance but was small in magnitude. This finding is consistent with its pharmacologic action on tear-film stability rather than tear volume. However, the clinical relevance of this small effect size remains uncertain [35]. Individual clinical trials show that although early outcomes are comparable to Cyclosporine, patients using Diquafosol often report greater comfort and visual quality with continued use, particularly beyond the first month of therapy [27,29]. Moreover, Jun et al. have demonstrated more pronounced improvements in symptoms and TBUT at around three months with patients using Diquafosol, supporting the concept of the cumulative mucin-mediated effect on tear homeostasis [36].

Previous studies were consistent with our finding that Diquafosol 3% is more advantageous for patients with severe DED than Cyclosporine. However, the interaction test between subgroups was not statistically significant (*p* = 0.3955), indicating that this finding should be interpreted as exploratory and hypothesis-generating rather than definitive.

According to previous observations, Diquafosol showed enhancements in ocular surface hydration by secreting mucin, which provides the damaged ocular surface with mechanical enhancement [37]. In severe cases, when the corneal epithelium is extensively damaged and there is substantial inflammation; this rapid stabilization of the tear film that Diquafosol probably provides more perceptible improvement to the patient than the slower anti-inflammatory activity of Cyclosporine [38]. Nakamura’s trial showed that Diquafosol rapidly elevates tear meniscus and improves conjunctival staining, which is a functional improvement that is especially useful for severely symptomatic dry ocular surfaces [34]. 

This meta-analysis, consistent with numerous RCTs, found no significant difference in TBUT between the two assessed interventions. For instance, Lee et al.’s trial found that both Diquafosol and Cyclosporine were effective after cataract surgery; however, Diquafosol showed a faster improvement in TBUT in the first month, while Cyclosporine’s benefits were more noticeable in reducing optical aberrations over three months [27]. Integrating data from multiple research studies indicates that TBUT may lack the sensitivity required to differentiate the unique physiological effects of these drugs. Lu et al. recently demonstrated that adding either Diquafosol or Cyclosporine to traditional postoperative therapy significantly enhanced tear film stability and lowered inflammatory cytokines compared to conventional treatment alone [28].

According to our findings, no substantial differences in TBUT or fluorescein staining between the two groups were demonstrated. This means that both medications might be equally effective at restoring the health of the ocular surface over a three-month period. The differences in the timing of symptom alleviation and tear generation are the primary factors that differentiate the two drugs from a clinical perspective.

Both medications showed comparable safety profiles regarding adverse events. Hence, clinicians can determine either to administer Cyclosporine and Diquafosol according to the specific needs of each patient, rather than to evade their side effects. Patients used to report stinging and burning sensations using previous Cyclosporine formulations; however, the development of advanced cationic emulsions and nanoemulsions has significantly improved the patient experience [39,40]. Diquafosol, on the other hand, is generally well-tolerated but needs to be taken more often (up to six times a day). These limitations can make it harder for patients to adhere to their treatment plan compared to the newer formulations of Cyclosporine, which only needs to be taken twice a day [41].

### 4.3. Strengths and Limitations 

To our knowledge, this is the first systematic review and meta-analysis comparing Diquafosol with Cyclosporine A in a head-to-head comparison for patients with DED. Our study combined the data of 859 patients from sex different RCTs and subgrouped the pooled analysis to increase the practical application of the findings in a clinical setting. We integrated TSA to avoid random errors often found in cumulative data. In addition, using Baujat plots effectively identified outlier studies that significantly influenced the overall results. Our study had numerous limitations. First of all, there was a considerable statistical heterogeneity, which was not resolved with leave-one-out meta-analysis across several outcomes. Such variability was mainly due to the differences in trial design, patient demographics, or the specific individual formulations. Among the included studies, one trial included patients with mild to moderate DED. TSA also pointed out that for the Corneoconjunctival Fluorescein Staining outcome, the total data have not yet reached the quantity required to be considered definite. As a result, the comparative effectiveness for this specific marker remains uncertain and inadequate. Moreover, major asymmetries were found in certain subgroups using Doi plot analysis, which suggests that there is a higher chance of publication bias or small-study effects. Non-English studies were excluded due to a lack of translation resources. This may have introduced bias. Also, retrospective registration of our study’s protocol should be considered among the limitations of the study.

Notably, none of the included studies were judged as having a high overall risk of bias. However, four studies had some concerns, primarily due to the lack of blinding of participants and personnel, a challenge inherent to topical eye drop trials where the active and comparator drops have different dosing frequencies (Cyclosporine A twice daily vs. Diquafosol sodium six times daily). This lack of blinding may introduce bias in patient-reported outcomes (e.g., symptom scores), but it is less likely to affect objective measurements such as TBUT and Schirmer test, which are performed by masked examiners using standardized procedures. The included studies varied substantially in baseline disease severity (mild-to-moderate-to-severe), populations (post-cataract surgery vs. chronic DED), and treatment regimens (dosing frequency). This heterogeneity limits the generalizability of our findings, and the results should be interpreted with caution when applied to specific DED subtypes.

### 4.4. Clinical Implications and Future Recommendations 

According to our findings, Cyclosporine A 0.05% and Diquafosol 3% are both effective and safe options for managing DED. However, Cyclosporine A appears to be a plausible choice for patients requiring a rapid increase in tear production, as evidenced by significantly improved Schirmer test results at the four-week mark. Conversely, Diquafosol 3% may be more effective specifically for improving symptom scores in patients with severe disease. To overcome current complications in the data, specifically high statistical heterogeneity and underpowered results, future research should prioritize large-scale, multi-center studies to confirm these findings with greater certainty.

Cyclosporine A is administered twice daily, while Diquafosol sodium requires six times daily administration in most studies. Lower dosing frequency is associated with better medication adherence in chronic eye disease. The frequent dosing schedule of Diquafosol may pose practical challenges for working patients or the elderly, although it offers better tolerability with minimal stinging. Clinicians should consider patient lifestyle and adherence capacity when selecting therapy.

Future studies should standardize their protocols regarding this topic and stratify patients by DED subtypes. This will aid in addressing the heterogeneity reported in our study outcomes. Moreover, we suggest future trials to prolong follow-up periods beyond twelve weeks to better capture the long-term therapeutic benefits of these two medicines. Finally, combination therapy using both a secretagogue and an immunomodulator remains insufficiently studied and warrants well-designed randomized trials to evaluate potential synergistic efficacy with careful safety monitoring.

## 5. Conclusions

Cyclosporine A was associated with a clinically modest improvement in Schirmer test at 4 weeks, while Diquafosol sodium was associated with a modest improvement in symptom scores at 12 weeks. Subgroup analysis suggested a potential benefit of Diquafosol sodium in severe disease; however, subgroup interaction tests were not statistically significant, and these findings should be considered exploratory. Given the heterogeneity and asymmetry found in our study, we recommend that future studies unify their protocols and extend their follow-up period beyond 12 weeks.

## Figures and Tables

**Table 1:** Summary of included studies.

Study ID	Study Design	Country	Population	Sample Size	Cyclosporine A Regimen	Diquafosol Sodium Regimen	Follow-Up	Key Findings
**Jung et al. 2023 [26]**	RCT	South Korea	Adults (≥19 years) with moderate-to-severe DED.	80	0.05% Cyclosporine A (Restasis) twice daily	3% Diquafosol (Diquas) six times daily	12 weeks	0.05% Cyclosporine A group showed significantly greater improvement in corneal erosion scores compared to the Diquafosol sodium group.
**Lee et al. 2016 [27]**	RCT	South Korea	Patients newly diagnosed with mild-to-moderate dry eye 1 week after cataract surgery.	40	0.05% Cyclosporine A twice daily	3.0% Diquafosol six times daily	12 weeks	Diquafosol sodium showed significantly better TBUT results than Cyclosporine A at 1 and 3 months.
**Lu et al. 2025 [28]**	RCT	China	Adults (≥29 years) with moderate-to-severe DED.	180	0.05% Cyclosporine A (Restasis) twice daily	3% Diquafosol sodium (Diquas) six times daily	12 weeks	At 12 weeks, Diquafosol sodium showed significantly better corneal staining scores and OSDI scores compared to Cyclosporine A.
**Park et al. 2019 [29]**	RCT	South Korea	Adults (≥19 years) with moderate DED.	227	0.05% Cyclosporine A Emulsion (Restasis) twice daily	3% Diquafosol sodium (Diquas) six times daily	12 weeks	Both groups showed significant improvements in corneal/conjunctival staining, TBUT, Schirmer, and OSDI.
**Parmar et al. 2024 [30]**	RCT	India	Adults (18–70 years) with moderate DED	50	0.05% topical Cyclosporin twice daily	3% Diquafosol sodium twice daily	12 weeks	Both groups showed significant improvements in TBUT and corneoconjunctival staining at 4 and 12 weeks.
**Xu et al. 2024 [25]**	RCT	China	College students (≥18 years) with moderate-to-severe DED	282	0.05% Cyclosporine A (Restasis) twice daily	3% Diquafosol sodium (Diquas) six times daily	12 weeks	Both groups showed significant improvements in corneal staining, OSDI, Schirmer, and TBUT at 12 weeks.

**DED:** Dry Eye Disease; **OSDI:** Ocular Surface Disease Index; **RCT:** randomized controlled trial; **TBUT:** Tear Break-Up Time.

**Table 2:** Baseline characteristics of included population.

Study ID	Group	Number	Age (Years), Mean (SD)	Female, N (%)	TBUT (Seconds), Mean (SD)	Schirmer Test, Mean (SD)	Corneal Staining Score, Mean (SD)	OSDI, Mean (SD)
**Jung et al. 2023 [26]**	Cyclosporine A	26	46.42 (17.05)	19 (73.08)	2.23 (1.26)	NA	NEI scale: 4.69 (1.12)	NA
	Diquafosol sodium	26	42.46 (17.15)	26 (100)	3.62 (1.08)	NA	NEI scale: 4.31 (0.93)	NA
**Lee et al. 2016 [27]**	Cyclosporine A	20	63.4 (12.2)	12 (60)	3.7 (1.08)	8.4 (3.6)	Oxford Grading System: 1.5 (0.83)	22.29 (13.66)
	Diquafosol sodium	20	64.3 (9.44)	12 (60)	3.17 (1.01)	7.7 (3.68)	Oxford Grading System: 1.41 (0.62)	20.1 (13.88)
**Lu et al. 2025 [28]**	Cyclosporine A	60	41.12 (6.61)	29 (48.33)	3.92 (0.82)	4.27 (1.08)	Oxford Grading System: 2.93 (0.23)	36.66 (11.41)
	Diquafosol sodium	60	42.55 (7.03)	30 (50)	4.21 (0.96)	4.59 (1.14)	Oxford Grading System: 3.01 (0.21)	37.34 (11.11)
**Park et al. 2019 [29]**	Cyclosporine A	58	NA	NA	3.89 (1.53)	7.90 (6.14)	NEI scale: 4.98 (1.42)	39.47 (18.96)
	Diquafosol sodium	57	NA	NA	4.29 (1.85)	7.96 (5.55)	NEI scale: 5.68 (1.84)	42.46 (18.9)
**Parmar et al. 2024 [30]**	Cyclosporine A	25	46.78 (6.79)	15 (60)	4.57 (1.11)	5.91 (2.99)	NEI scale: 5.22 (1.04)	46.64 (15.65)
	Diquafosol sodium	25	52.36 (7.92)	16 (64)	4.42 (0.93)	5.68 (3.59)	NEI scale: 4.67 (1.96)	47.26 (18.94)
**Xu et al. 2024 [25]**	Cyclosporine A	94	20.01 (1.97)	73 (77.66)	4.03 (1.65)	3.97 (1.78)	Oxford Grading System: 2.88 (0.49)	37.04 (20.13)
	Diquafosol sodium	94	20.51 (1.39)	74 (78.72)	3.94 (1.58)	4.29 (1.85)	Oxford Grading System: 2.95 (0.55)	36.61 (18.89)

**Cyclosporine A:** Cyclosporine A; **Diquafosol sodium:** Diquafosol sodium; **NEI:** National Eye Institute; **OSDI:** Ocular Surface Disease Index; **TBUT:** Tear Break-Up Time; NA: Not Available.

## Data Availability

Data available on request from the corresponding author.

## Supplementary Materials

The following supporting information can be downloaded at: https://www.mdpi.com/article/10.3390/jcm15124823/s1, Table S1. Search strategies used for all databases included in the systematic review. Table S2. PRISMA 2020 Checklist. Figure S1. Leave-one-out sensitivity analysis for Tear Break-Up Time (TBUT). Figure S2. Baujat plot for Tear Break-Up Time (TBUT) at 12 weeks. Figure S3. Doi plot for Tear Break-Up Time (TBUT) at 12 weeks. Figure S4. Doi plot for Corneoconjunctival Fluorescein Staining at 12 weeks. Figure S5. Trial Sequential Analysis (TSA) for Corneoconjunctival Fluorescein Staining at 12 weeks. Figure S6. Leave-one-out sensitivity analysis for Corneoconjunctival Fluorescein Staining. Figure S7. Leave-one-out sensitivity analysis for Corneal Fluorescein Staining. Figure S8. Leave-one-out sensitivity analysis for Conjunctival Fluorescein Staining. Figure S9. Leave-one-out sensitivity analysis for Schirmer Test. Figure S10. Leave-one-out sensitivity analysis for Symptom Scores. Figure S11. Doi plot for Schirmer Test at 12 weeks. Figure S12. Doi plot for Symptom Scores at 12 weeks. Figure S13. Baujat plot for Schirmer Test at 12 weeks. Figure S14. Baujat plot for Symptom Scores at 12 weeks. Figure S15. Trial Sequential Analysis (TSA) for Tear Break-Up Time (TBUT) at 12 weeks. Figure S16. Trial Sequential Analysis (TSA) for Schirmer Test at 12 weeks. Figure S17. Trial Sequential Analysis (TSA) for Symptom Scores at 12 weeks. Figure S18. Publication bias assessment for Tear Break-Up Time (TBUT). Figure S19. Additional sensitivity and heterogeneity analyses for the primary outcomes.
