# Supplementary material for: Comparative Efficacy and Safety of 0.05% Cyclosporine A and 3% Diquafosol Sodium in Dry Eye Disease: A Systematic Review and Meta-Analysis with Trial Sequential Analysis
Source: J Clin Med. 2026 Jun 21;15(12):4823. doi: 10.3390/jcm15124823 (PMC13302226; doi:10.3390/jcm15124823)
Supplement: Supplementary file 1 [file jcm-15-04823-s001.zip › jcm-4280842-supplementary.pdf]

Supplementary Appendix

Table of Contents

Page	Item
2	**Table S1.** Search Strategies
3	**Figure S1.** Leave-one-out meta-analysis for TBUT
4	**Figure S2.** Baujat plot for Tear Break-Up Time (TBUT) at 12 weeks
5	**Figure S3.** Doi plot for Tear Break-Up Time (TBUT) at 12 weeks
6	**Figure S4.** Doi plot for Corneoconjunctival Fluorescein Staining at 12 weeks
7	**Figure S5.** Trial Sequential Analysis (TSA) graph for Corneoconjunctival Fluorescein Staining at 12 weeks
8	**Figure S6.** Baujat plot for Corneal Fluorescein Staining at 12 weeks
9	**Figure S7.** Doi plot for Corneal Fluorescein Staining at 12 weeks
10	**Figure S8.** Doi plot for Conjunctival Fluorescein Staining at 12 weeks
11	**Figure S9.** Leave-one-out meta-analysis for Schirmer Test at 12 weeks
12	**Figure S10.** Doi plot for Schirmer Test at 12 weeks
13	**Figure S11.** Doi plot for Symptom Scores at 12 weeks
14	**Figure S12.** Doi plot for Adverse Events
15	**Figure S13.** Leave-one-out meta-analysis for TBUT Subgroup Analysis by Disease Severity
16	**Figure S14.** Baujat plot for TBUT in Severe Disease Subgroup
17	**Figure S15.** Doi plot for TBUT in Severe Disease Subgroup
18	**Figure S16.** Funnel plot after trim-and-fill meta-analysis for TBUT in Severe Disease Subgroup
19	**Figure S17.** Leave-one-out meta-analysis for Schirmer Test in Mild-to-Moderate/Moderate Subgroup
20	**Figure S18.** Doi plot for Schirmer Test in Severe Disease Subgroup
21	**Figure S19.** Doi plot for Symptom Scores in Severe Disease Subgroup
22-23	**Table S2. PRISMA 2020 Checklist**

Table S1. Search strategies

Databases	Search strategies	Results	Limitations	Date of search

**PubMed**	("dry eye*" OR "keratoconjunctivitis sicca" OR "ocular surface disease") AND (cyclosporin* OR CsA OR restasis OR "Immunosuppressive eye drops" OR Ikervis) AND (Diquafosol OR "P2Y2 receptor agonist" OR diquas OR INS365 OR "INS 365") (All fields)	36	No limitations applied	21 January 2026
**Web of science**	("dry eye*" OR "keratoconjunctivitis sicca" OR "ocular surface disease") AND (cyclosporin* OR CsA OR restasis OR "Immunosuppressive eye drops" OR Ikervis) AND (Diquafosol OR "P2Y2 receptor agonist" OR diquas OR INS365 OR "INS 365") (All fields)	65	No limitations applied	21 January 2026
**Cochrane library**	("dry eye*" OR "keratoconjunctivitis sicca" OR "ocular surface disease") AND (cyclosporin* OR CsA OR restasis OR "Immunosuppressive eye drops" OR Ikervis) AND (Diquafosol OR "P2Y2 receptor agonist" OR diquas OR INS365 OR "INS 365") (All text)	14	No limitations applied	21 January 2026
**Scopus**	("dry eye*" OR "keratoconjunctivitis sicca" OR "ocular surface disease") AND (cyclosporin* OR CsA OR restasis OR "Immunosuppressive eye drops" OR Ikervis) AND (Diquafosol OR "P2Y2 receptor agonist" OR diquas OR INS365 OR "INS 365") (Title, Abstract, Keywords)	142	No limitations applied	21 January 2026

Study	SMD	95%-CI	P-value	Tau2	Tau	I2
Omitting Jung et al. 2023	-0.04	[-0.35; 0.26]	0.7903	0.0732	0.2706	59.7%
Omitting Lee et al. 2016	0.08	[-0.09; 0.25]	0.3428	0	0	0%
Omitting Lu et al. 2025	-0.03	[-0.38; 0.33]	0.8814	0.1039	0.3224	61.9%
Omitting Park et al. 2019	-0.06	[-0.37; 0.26]	0.7275	0.0725	0.2692	56.3%
Omitting Parmar et al. 2024	-0.04	[-0.35; 0.27]	0.8120	0.0769	0.2773	60.4%
Omitting Xu et al. 2024	0.00	[-0.36; 0.36]	0.9805	0.1065	0.3263	60.5%
**Random effects model**	**0.01**	**[-0.23; 0.25]**	**0.9629**	**0.0397**	**0.1992**	**52.8%**

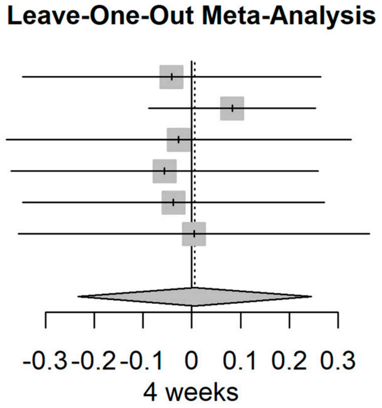

Study	SMD	95%-CI	P-value	Tau2	Tau	I2
Omitting Jung et al. 2023	-0.20	[-0.62; 0.22]	0.3566	0.1772	0.4209	71.4%
Omitting Lee et al. 2016	0.10	[-0.17; 0.38]	0.4642	0.0564	0.2374	56.8%
Omitting Lu et al. 2025	-0.06	[-0.66; 0.54]	0.8404	0.4077	0.6385	82.1%
Omitting Park et al. 2019	-0.12	[-0.71; 0.46]	0.6844	0.3804	0.6168	80.5%
Omitting Parmar et al. 2024	-0.08	[-0.67; 0.51]	0.7928	0.3953	0.6287	82.2%
Omitting Xu et al. 2024	-0.05	[-0.65; 0.56]	0.8797	0.4072	0.6381	81.4%
**Random effects model**	**-0.06**	**[-0.53; 0.41]**	**0.8039**	**0.2887**	**0.5373**	**77.7%**

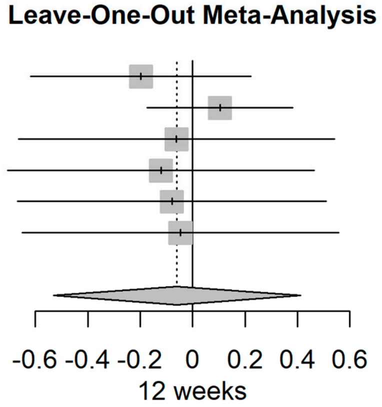

Figure S1: Leave one out meta-analysis for Subgroup analysis by follow up time for Change in TBUT, (sec)

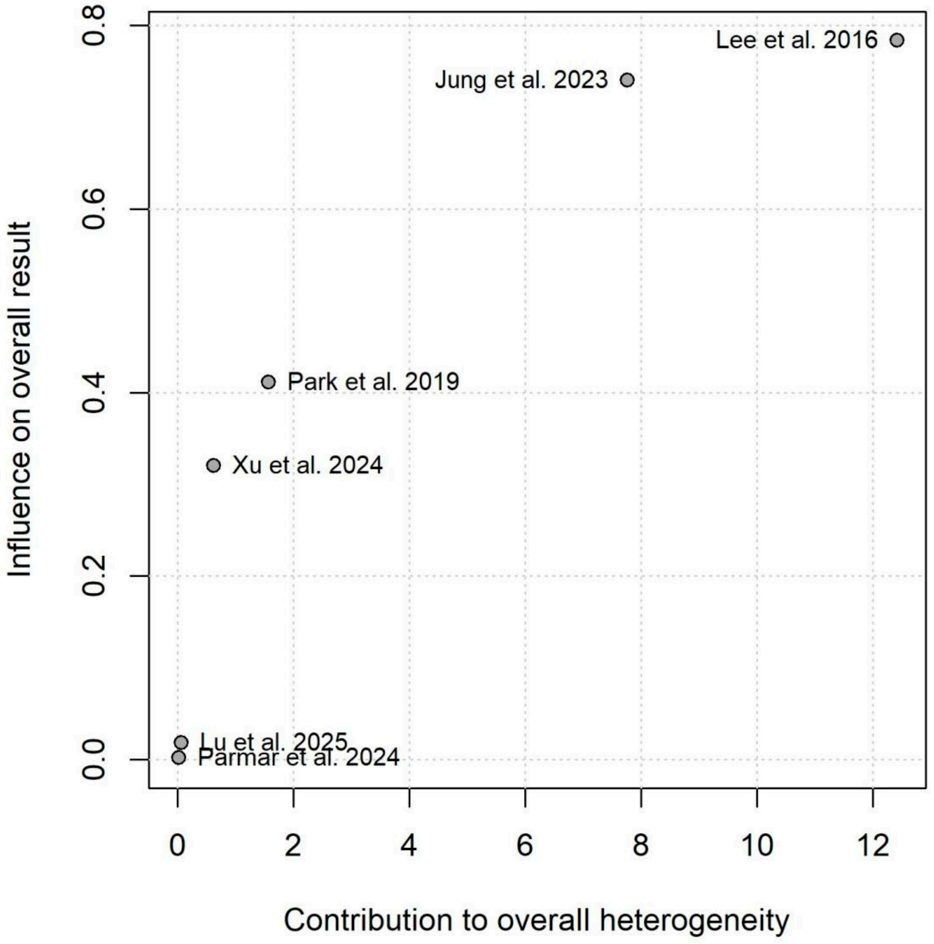

Figure S2: Baujat plot for Change in Tear Break-Up Time (TBUT), (sec) at 12 weeks

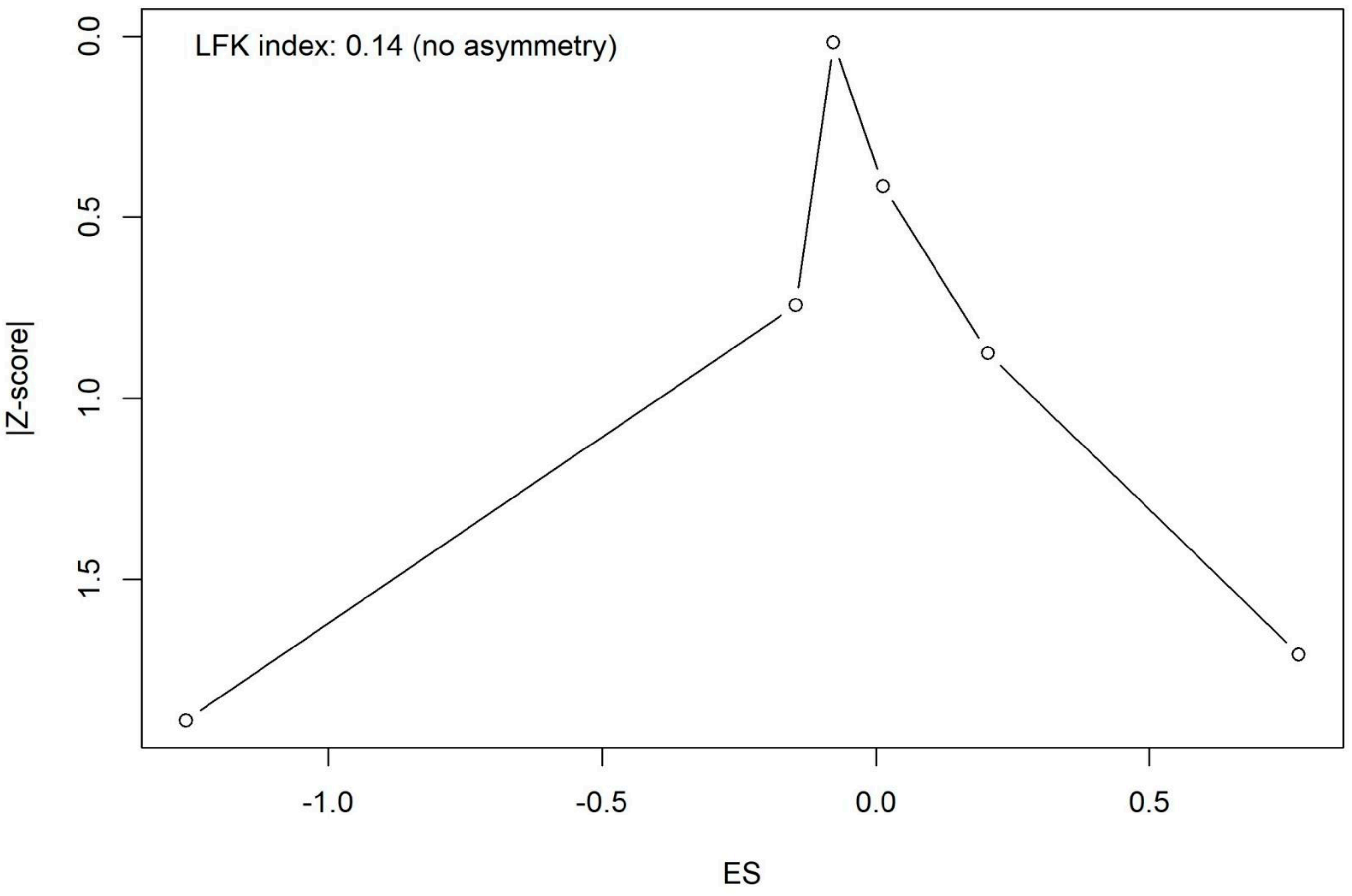

Figure S3: Doi plot for Change in Tear Break-Up Time (TBUT), (sec) at 12 weeks

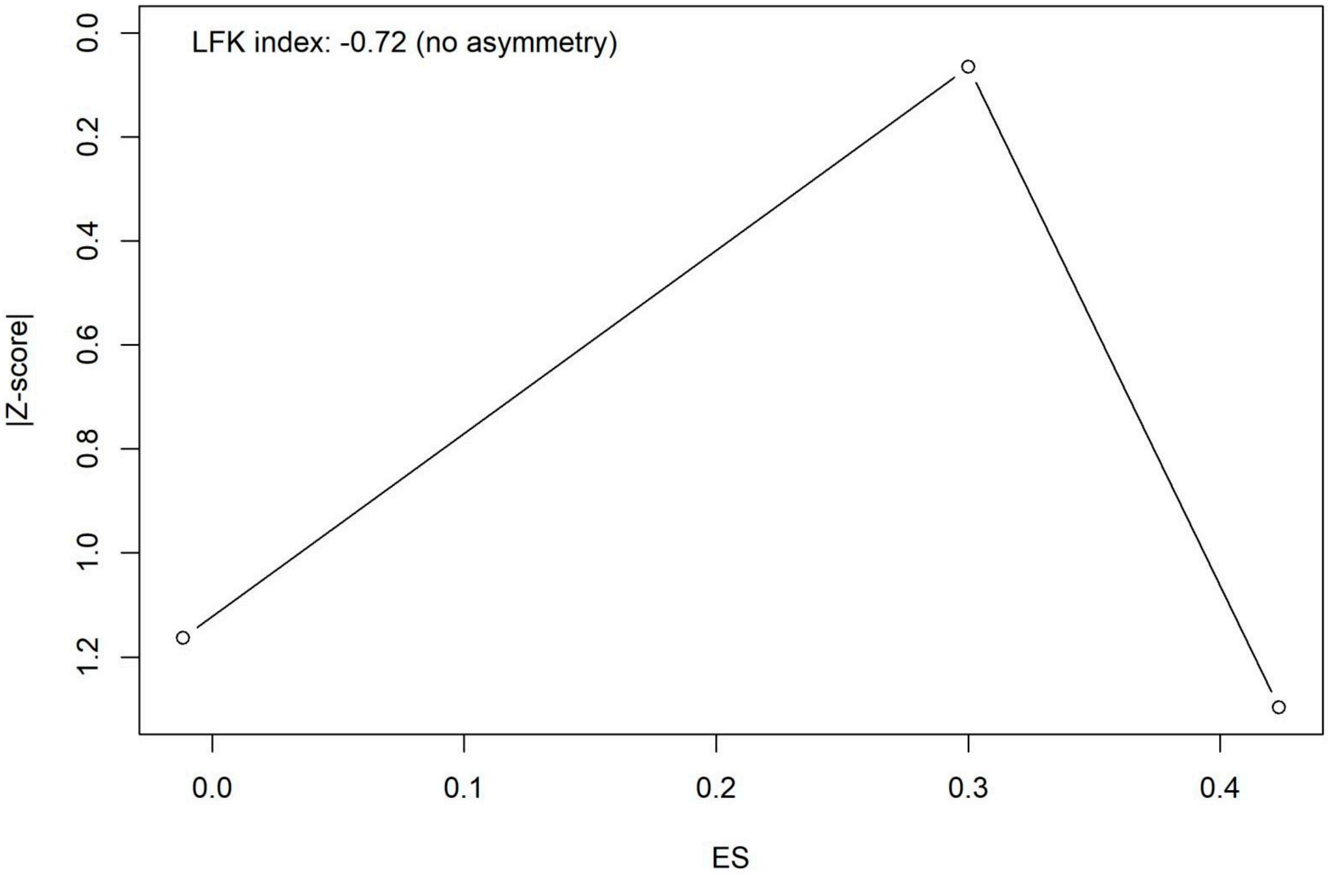

Figure S4: Doi plot for Change in Corneoconjunctival Fluorescein Staining at 12 weeks

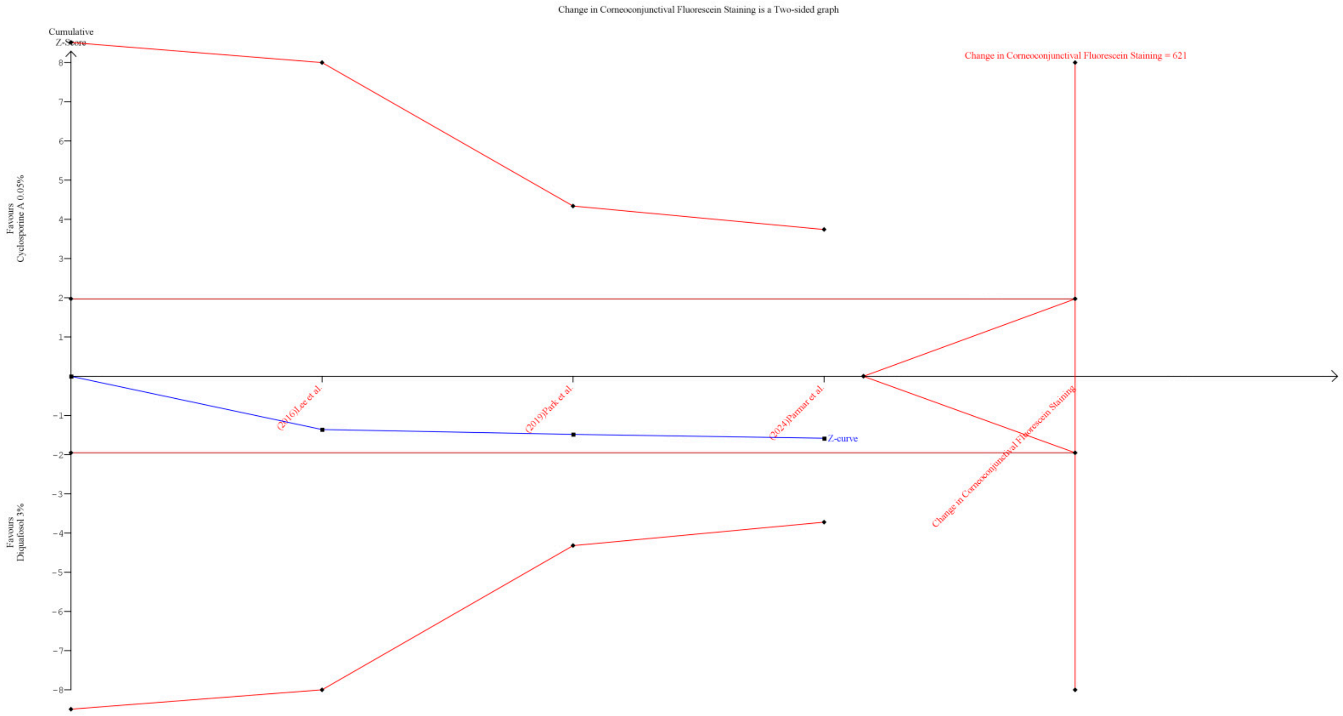

Figure S5: TSA graph for Change in Corneoconjunctival Fluorescein Staining at 12 weeks

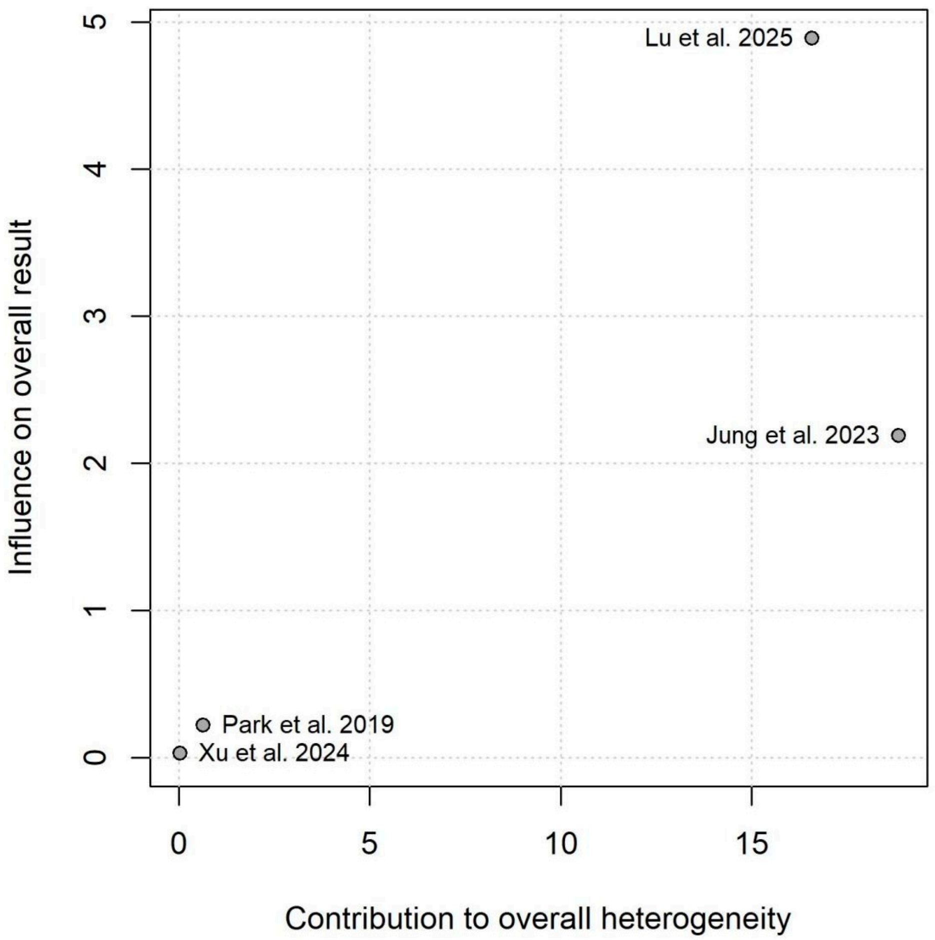

Figure S6: Baujat plot for Change in Corneal Fluorescein Staining at 12 weeks

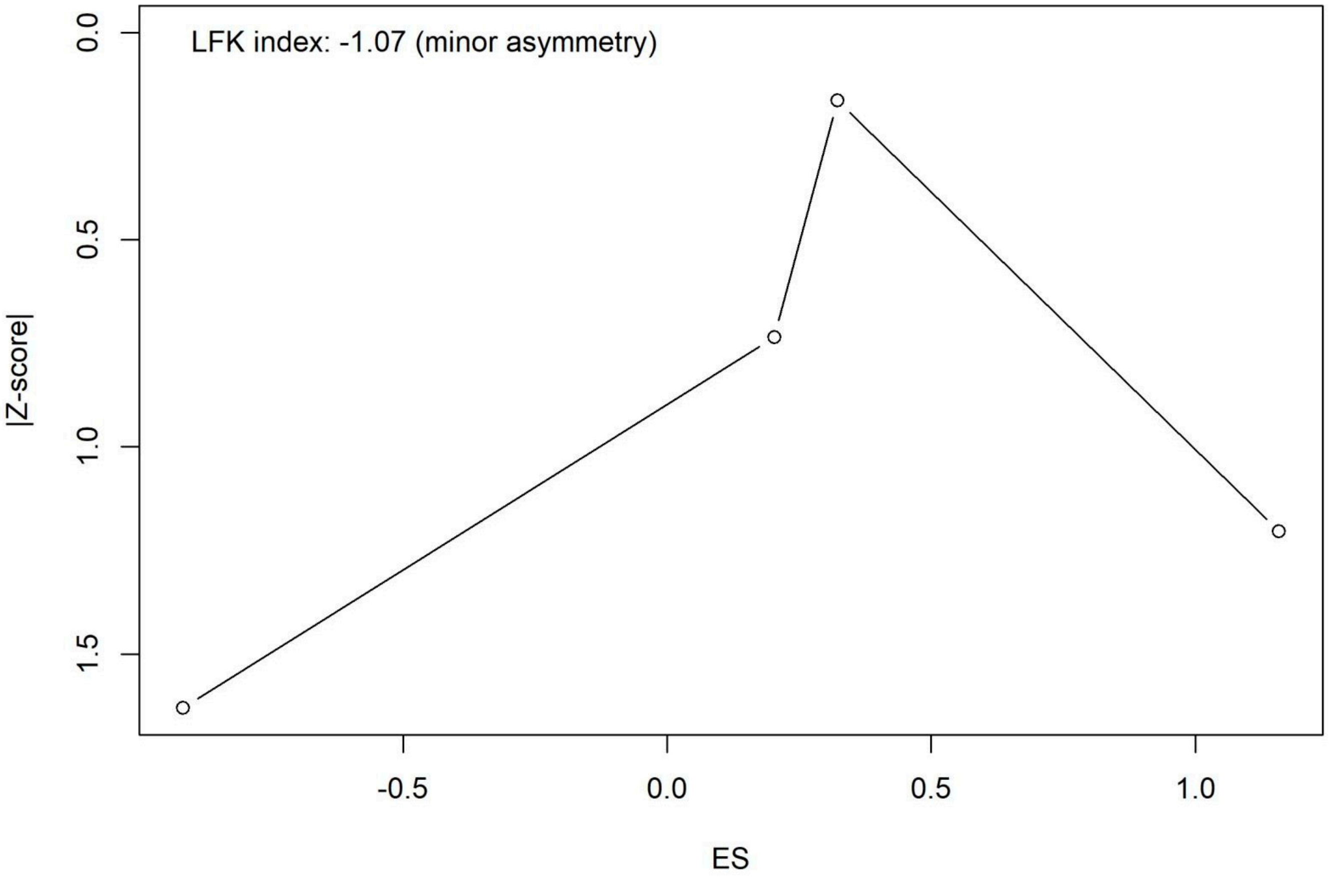

Figure S7: Doi plot for Change in Corneal Fluorescein Staining at 12 weeks

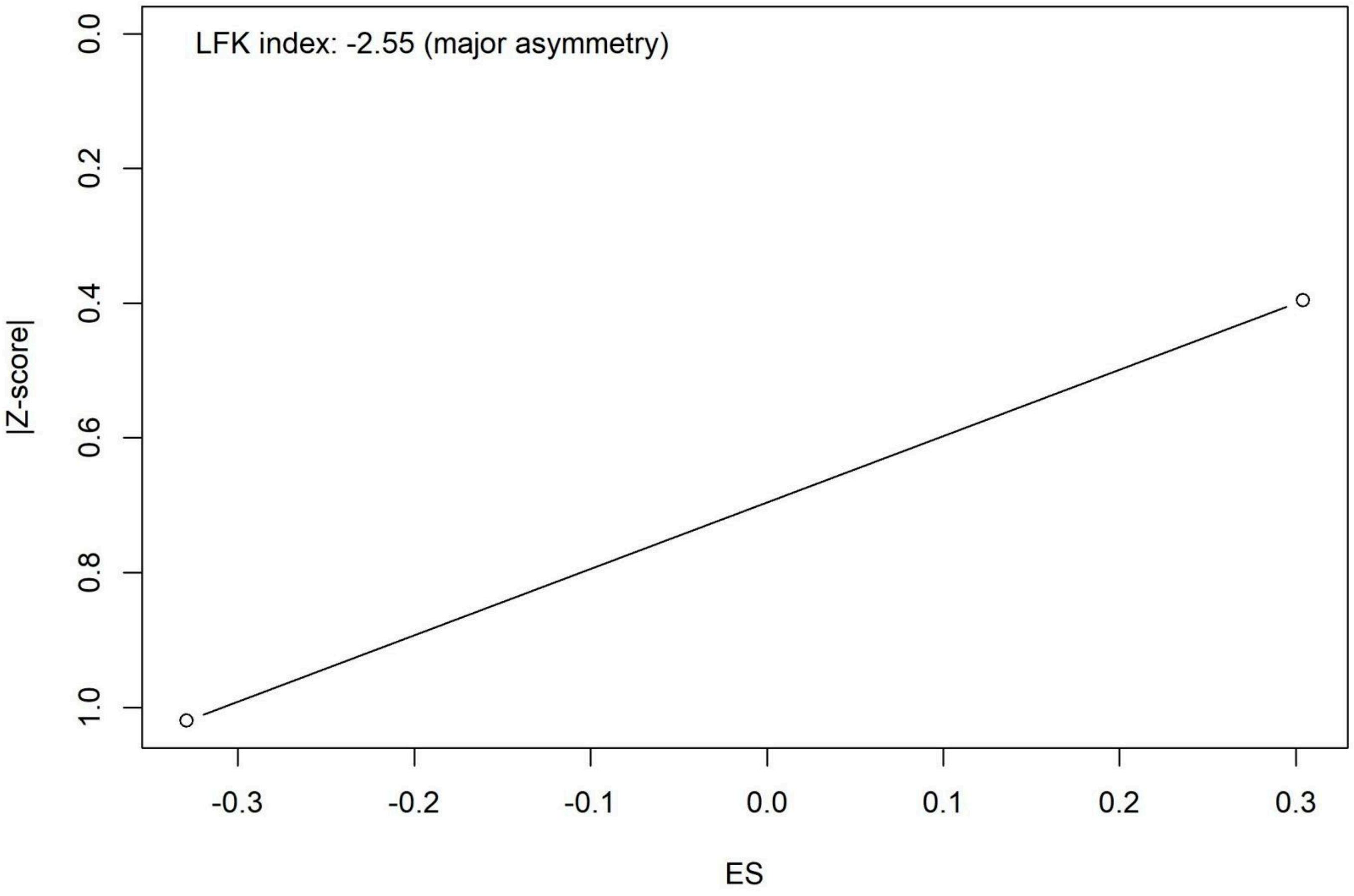

Figure S8: Doi plot for Change in Conjunctival Fluorescein Staining at 12 weeks

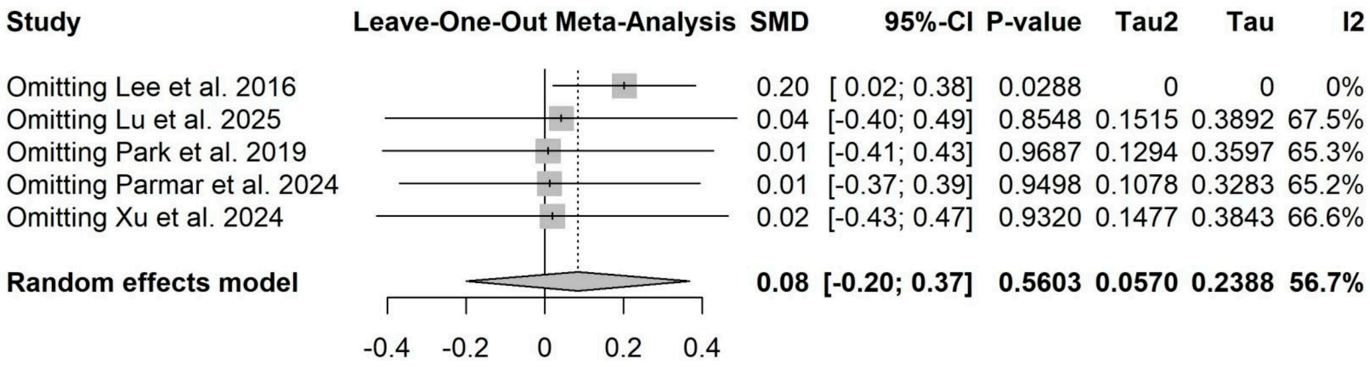

Figure S9: Leave one out meta-analysis for Change in Schirmer Test at 12 weeks

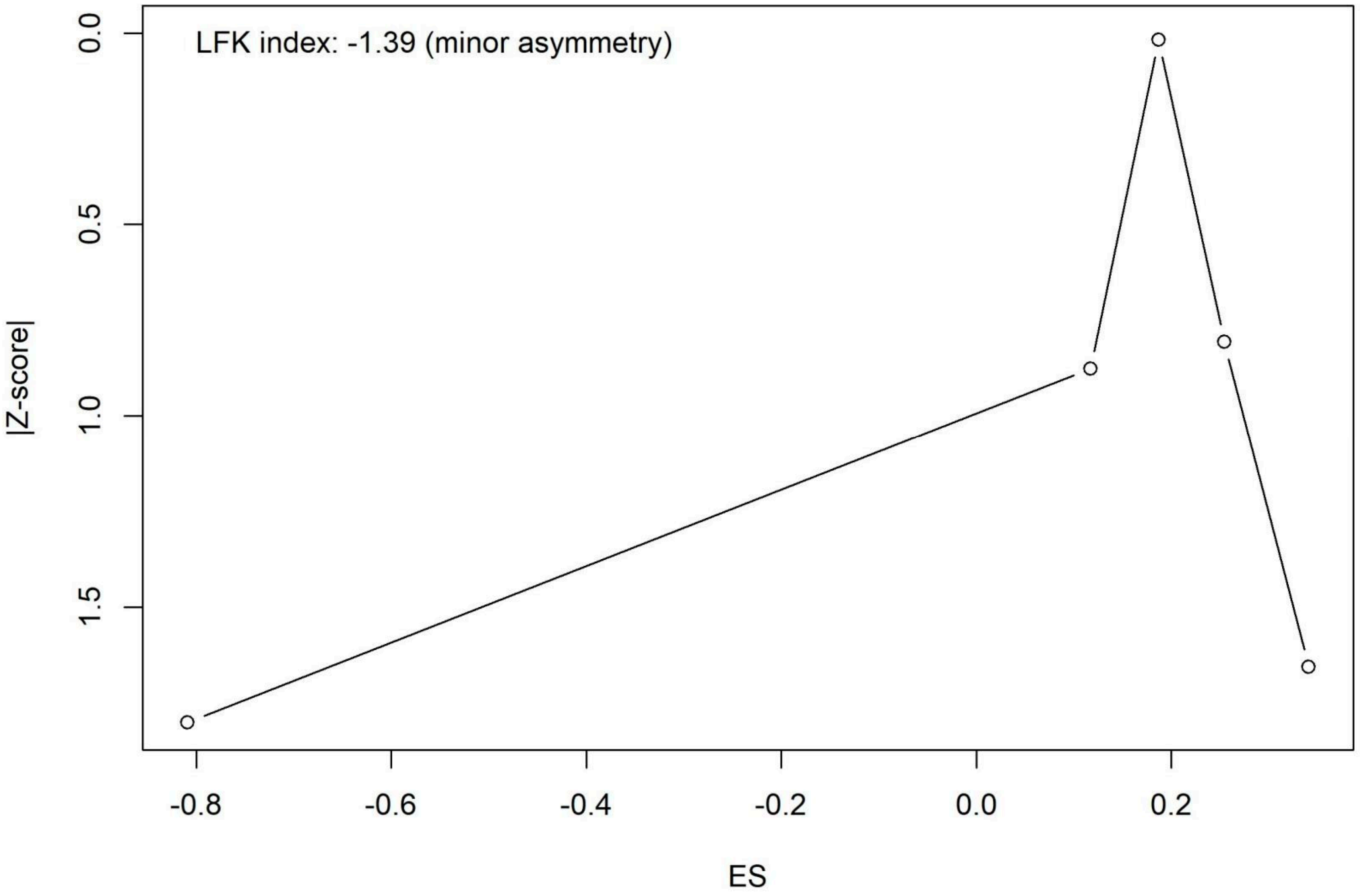

Figure S10: Doi plot for Change in Schirmer Test at 12 weeks

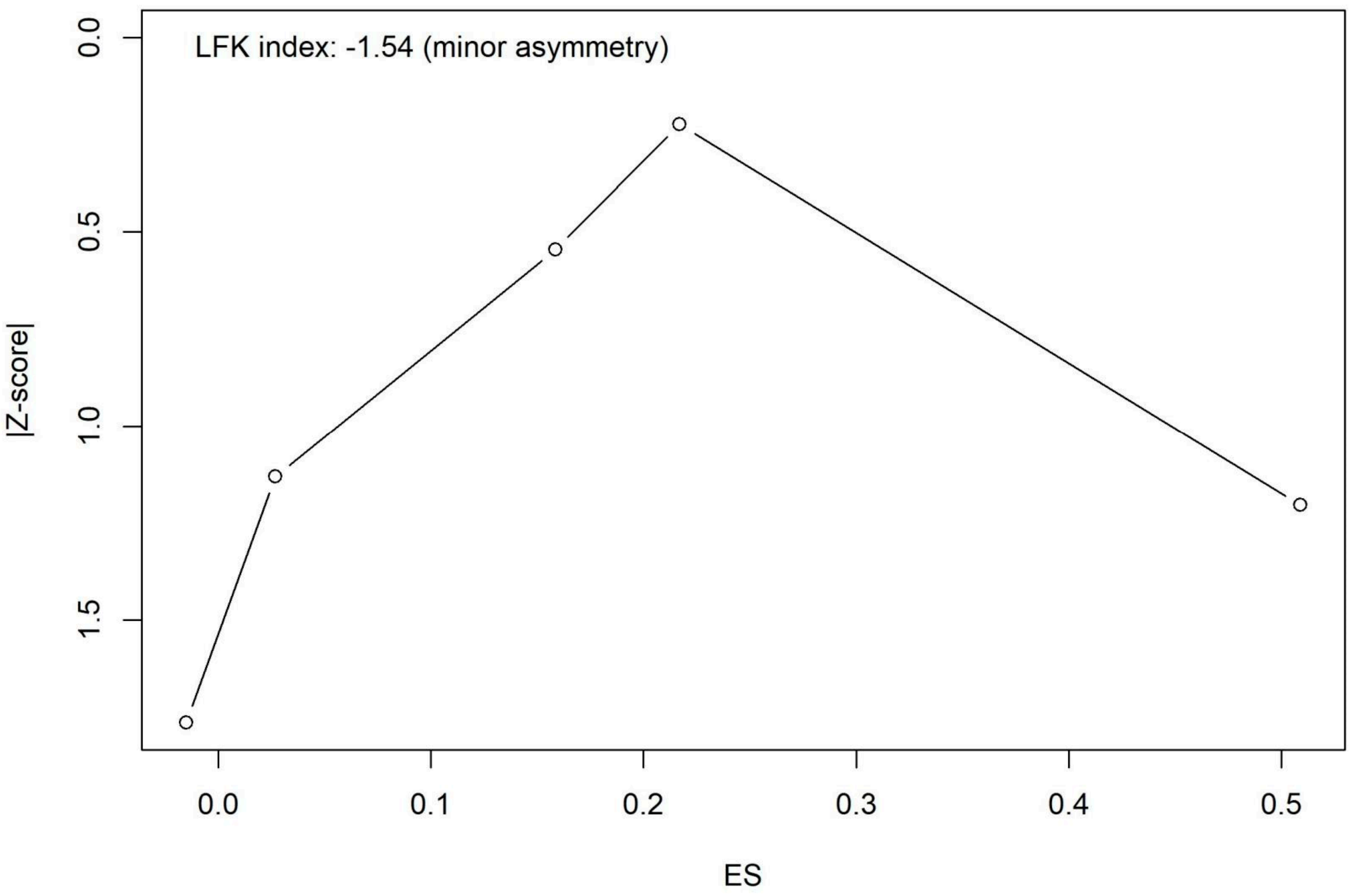

Figure S11: Doi plot for Change in Symptom Scores at 12 weeks

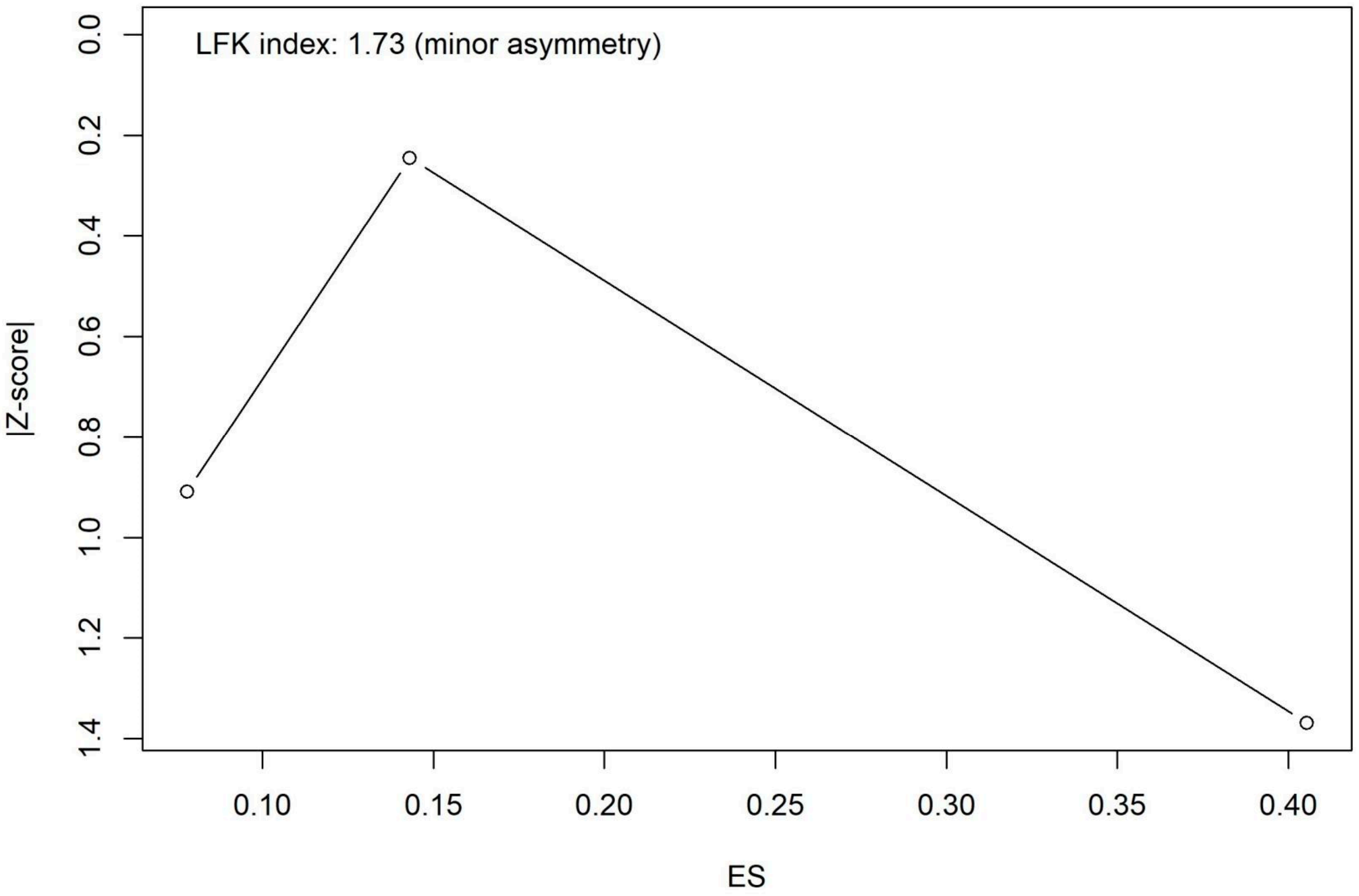

Figure S12: Doi plot for Adverse events

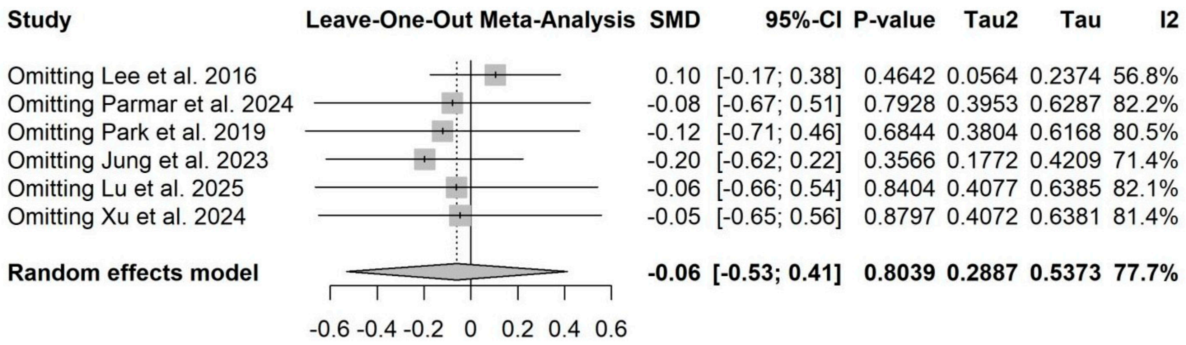

Figure S13: Leave one out meta-analysis for Subgroup analysis by disease severity for Change in Tear Break-Up Time (TBUT), (sec)

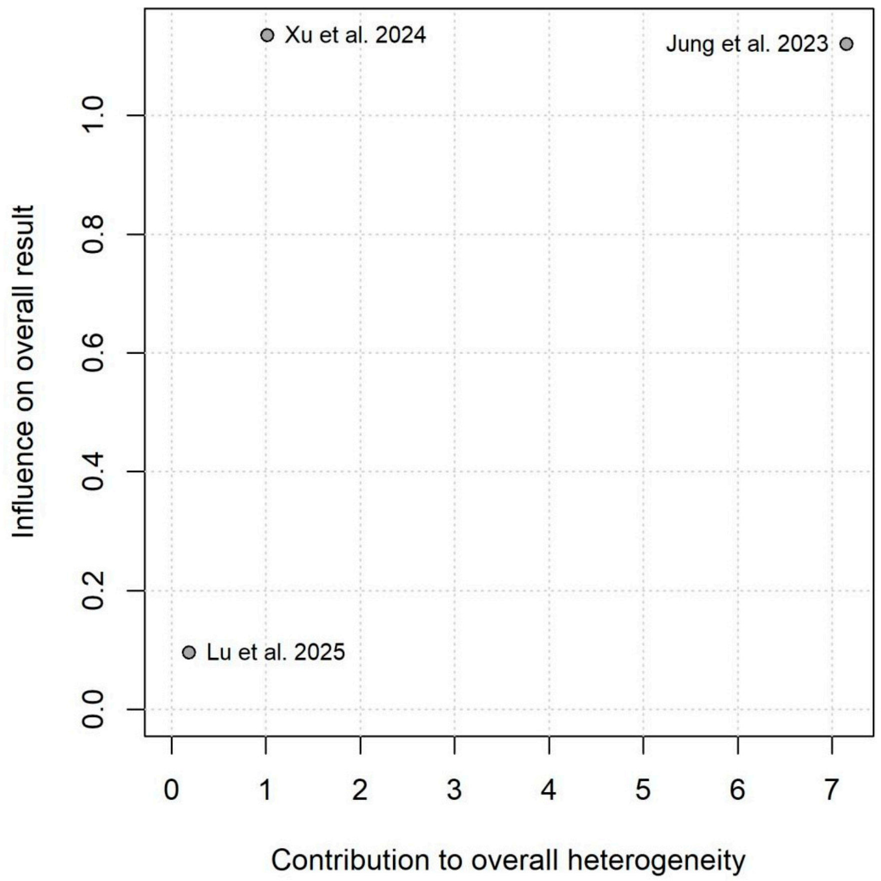

Figure S14: Baujat plot for Change in TBUT for sever TBUT

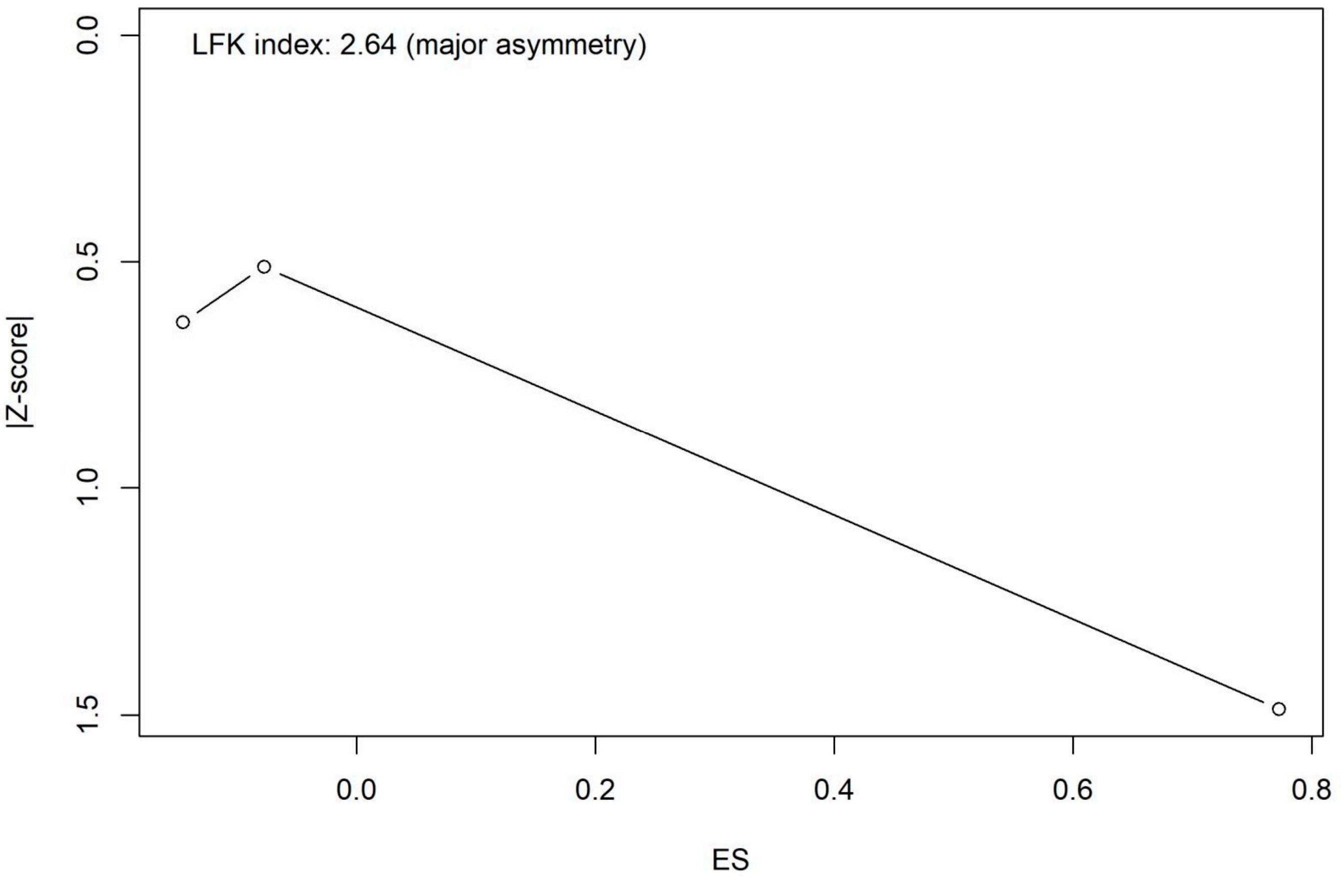

Figure S15: Doi plot for Change in TBUT for sever TBUT

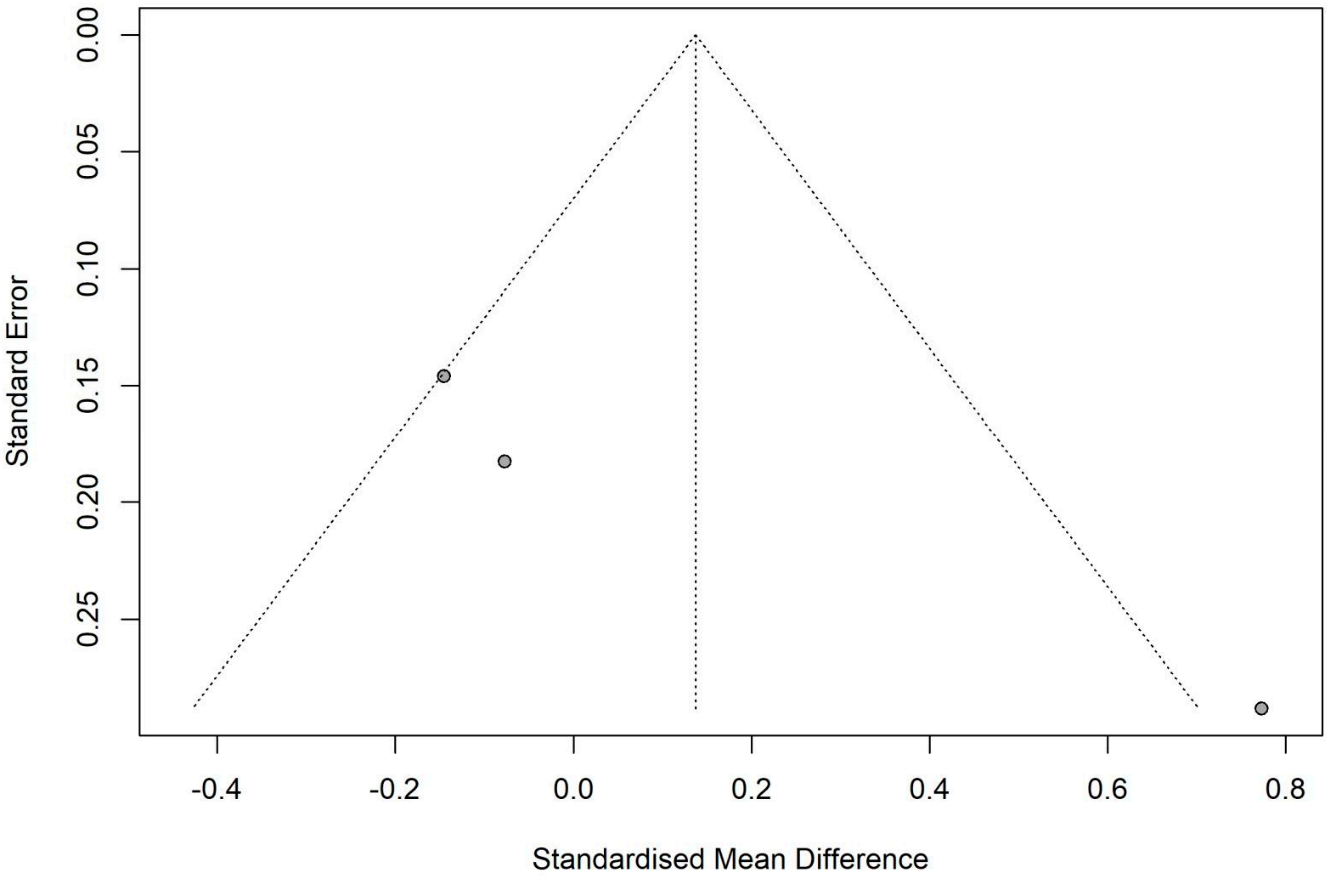

Figure S16: Funnel plot after trim and fill meta-analysis for for Change in TBUT for sever TBUT

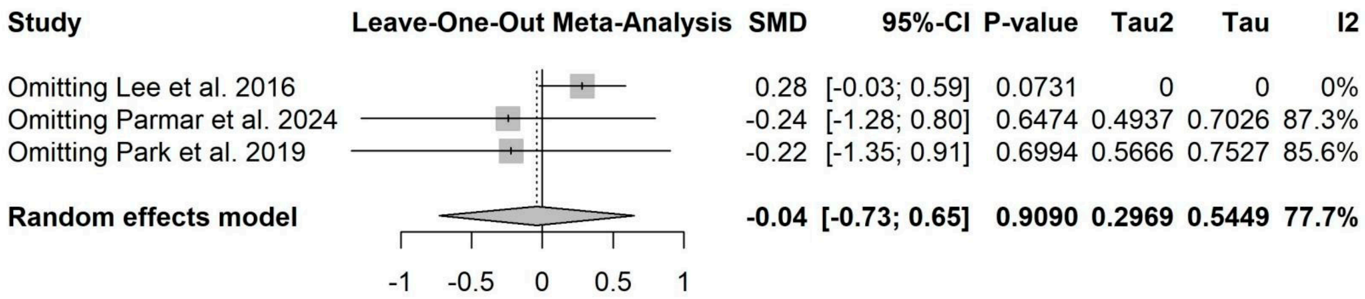

Figure S17: Leave one out meta-analysis for Change in Schirmer Test for mild-to-moderate/moderate subgroup

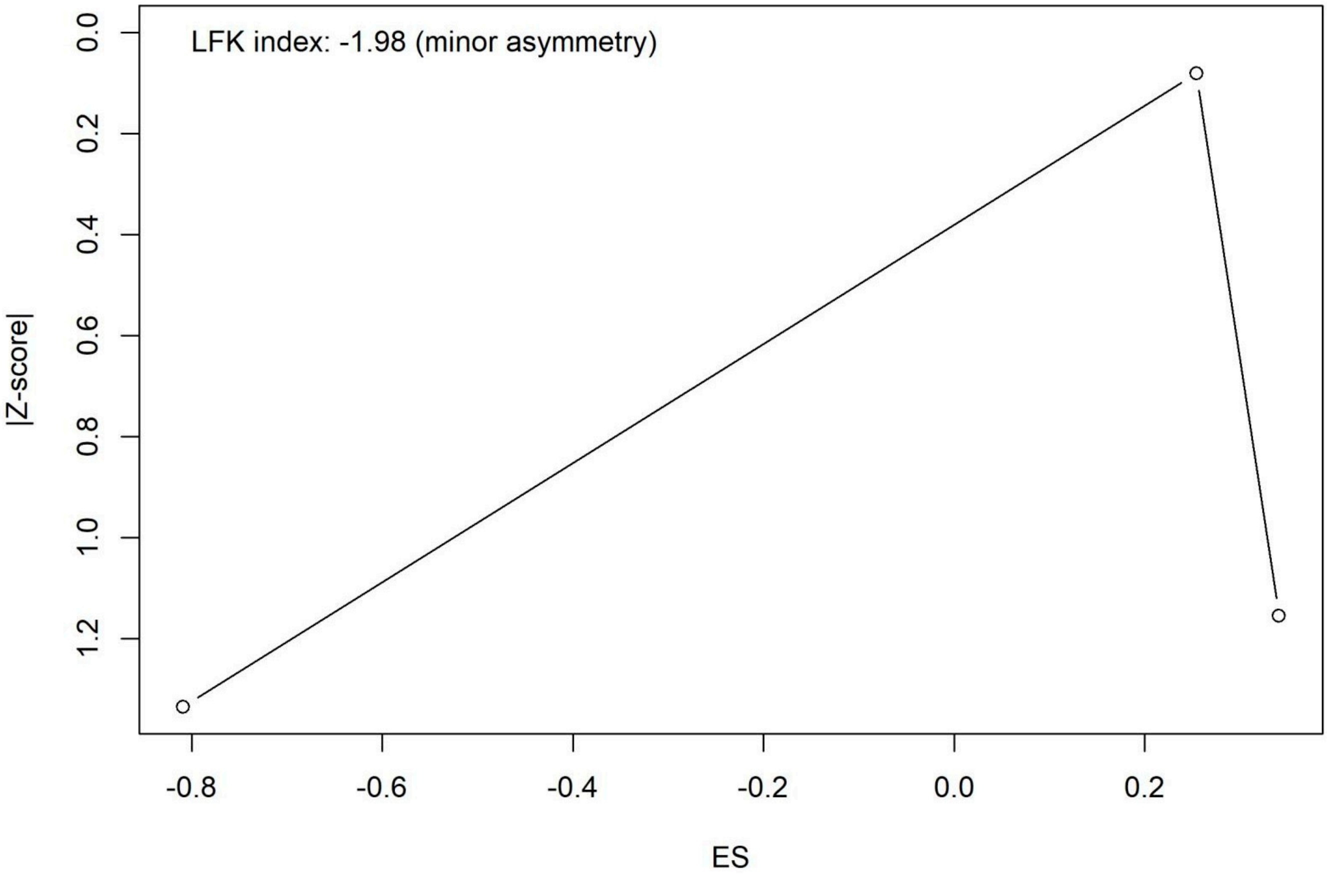

Figure S18: Doi plot for Change in Schirmer Test for Sever patients

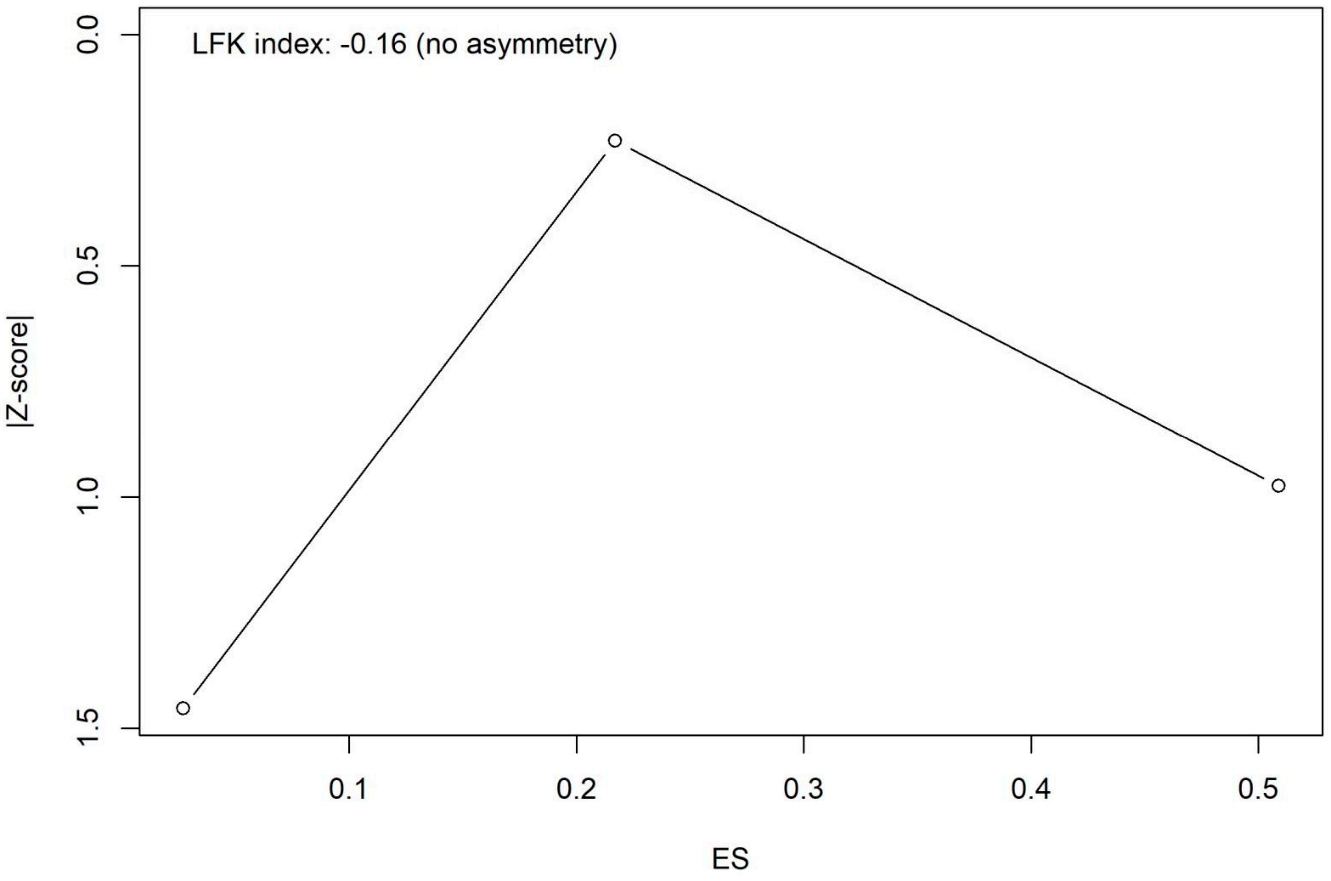

Figure S19: Doi plot for Change in Symptom Scores for severe patients

Table S2. PRISMA checklist

Section/topic	#	Checklist item	Reported on page #
**TITLE**			
Title	1	Identify the report as a systematic review, meta-analysis, or both.	1
**ABSTRACT**			
Structured summary	2	Provide a structured summary including, as applicable: background; objectives; data sources; study eligibility criteria, participants, and interventions; study appraisal and synthesis methods; results; limitations; conclusions and implications of key findings; systematic review registration number.	1
**INTRODUCTION**			
Rationale	3	Describe the rationale for the review in the context of what is already known.	2
Objectives	4	Provide an explicit statement of questions being addressed with reference to participants, interventions, comparisons, outcomes, and study design (PICOS).	2
**METHODS**			
Protocol and registration	5	Indicate if a review protocol exists, if and where it can be accessed (e.g., Web address), and, if available, provide registration information including registration number.	3
Eligibility criteria	6	Specify study characteristics (e.g., PICOS, length of follow-up) and report characteristics (e.g., years considered, language, publication status) used as criteria for eligibility, giving rationale.	3
Information sources	7	Describe all information sources (e.g., databases with dates of coverage, contact with study authors to identify additional studies) in the search and date last searched.	3
Search	8	Present full electronic search strategy for at least one database, including any limits used, such that it could be repeated.	3
Study selection	9	State the process for selecting studies (i.e., screening, eligibility, included in systematic review, and, if applicable, included in the meta-analysis).	3
Data collection process	10	Describe method of data extraction from reports (e.g., piloted forms, independently, in duplicate) and any processes for obtaining and confirming data from investigators.	3
Data items	11	List and define all variables for which data were sought (e.g., PICOS, funding sources) and any assumptions and simplifications made.	3
Risk of bias in individual studies	12	Describe methods used for assessing risk of bias of individual studies (including specification of whether this was done at the study or outcome level), and how this information is to be used in any data synthesis.	3-4
Summary measures	13	State the principal summary measures (e.g., risk ratio, difference in means).	4
Synthesis of results	14	Describe the methods of handling data and combining results of studies, if done, including measures of consistency (e.g., I^2) for each meta-analysis.	4

Section/topic	#	Checklist item	Reported on page #
Risk of bias across studies	15	Specify any assessment of risk of bias that may affect the cumulative evidence (e.g., publication bias, selective reporting within studies).	4

Additional analyses	16	Describe methods of additional analyses (e.g., sensitivity or subgroup analyses, meta-regression), if done, indicating which were pre-specified.	4
**RESULTS**			
Study selection	17	Give numbers of studies screened, assessed for eligibility, and included in the review, with reasons for exclusions at each stage, ideally with a flow diagram.	4-5
Study characteristics	18	For each study, present characteristics for which data were extracted (e.g., study size, PICOS, follow-up period) and provide the citations.	5-7
Risk of bias within studies	19	Present data on risk of bias of each study and, if available, any outcome level assessment (see item 12).	7
Results of individual studies	20	For all outcomes considered (benefits or harms), present, for each study: (a) simple summary data for each intervention group (b) effect estimates and confidence intervals, ideally with a forest plot.	9-15
Synthesis of results	21	Present results of each meta-analysis done, including confidence intervals and measures of consistency.	9-15
Risk of bias across studies	22	Present results of any assessment of risk of bias across studies (see Item 15).	9-15
Additional analysis	23	Give results of additional analyses, if done (e.g., sensitivity or subgroup analyses, meta-regression [see Item 16]).	9-15
**DISCUSSION**			
Summary of evidence	24	Summarize the main findings including the strength of evidence for each main outcome; consider their relevance to key groups (e.g., healthcare providers, users, and policy makers).	17-18
Limitations	25	Discuss limitations at study and outcome level (e.g., risk of bias), and at review-level (e.g., incomplete retrieval of identified research, reporting bias).	17
Conclusions	26	Provide a general interpretation of the results in the context of other evidence, and implications for future research.	18
**FUNDING**			
Funding	27	Describe sources of funding for the systematic review and other support (e.g., supply of data); role of funders for the systematic review.	19-20
